# Proline-rich transmembrane protein 2 (*PRRT2*) regulates the actin cytoskeleton during synaptogenesis

**DOI:** 10.1038/s41419-020-03073-w

**Published:** 2020-10-14

**Authors:** Elisa Savino, Romina Inès Cervigni, Miriana Povolo, Alessandra Stefanetti, Daniele Ferrante, Pierluigi Valente, Anna Corradi, Fabio Benfenati, Fabrizia Claudia Guarnieri, Flavia Valtorta

**Affiliations:** 1 IRCCS San Raffaele Scientific Institute, Via Olgettina 60, 20132 Milan, Italy; 2 Vita-Salute San Raffaele University, Via Olgettina 58, 20132 Milan, Italy; 3 Department of Experimental Medicine, University of Genova, Viale Benedetto XV, 3, 16132 Genova, Italy; 4 IRCCS Ospedale Policlinico San Martino, Largo Rosanna Benzi 10, 16132 Genova, Italy; 5 Center for Synaptic Neuroscience and Technology, Istituto Italiano di Tecnologia, Largo Rosanna Benzi 10, 16132 Genova, Italy

**Keywords:** Actin, Molecular neuroscience, Neurological disorders

## Abstract

Mutations in proline-rich transmembrane protein 2 (*PRRT2*) have been recently identified as the leading cause of a clinically heterogeneous group of neurological disorders sharing a paroxysmal nature, including paroxysmal kinesigenic dyskinesia and benign familial infantile seizures. To date, studies aimed at understanding its physiological functions in neurons have mainly focused on its ability to regulate neurotransmitter release and neuronal excitability. Here, we show that PRRT2 expression in non-neuronal cell lines inhibits cell motility and focal adhesion turnover, increases cell aggregation propensity, and promotes the protrusion of filopodia, all processes impinging on the actin cytoskeleton. In primary hippocampal neurons, PRRT2 silencing affects the synaptic content of filamentous actin and perturbs actin dynamics. This is accompanied by defects in the density and maturation of dendritic spines. We identified cofilin, an actin-binding protein abundantly expressed at the synaptic level, as the ultimate effector of PRRT2. Indeed, PRRT2 silencing unbalances cofilin activity leading to the formation of cofilin-actin rods along neurites. The expression of a cofilin phospho-mimetic mutant (cof-S3E) is able to rescue PRRT2-dependent defects in synapse density, spine number and morphology, but not the alterations observed in neurotransmitter release. Our data support a novel function of PRRT2 in the regulation of the synaptic actin cytoskeleton and in the formation of synaptic contacts.

## Introduction

Mutations in the *Proline-Rich Transmembrane protein 2* (*PRRT2*) gene were identified at the basis of a broad and heterogeneous spectrum of neurological conditions sharing a paroxysmal nature. This group includes paroxysmal movement disorders, such as paroxysmal kinesigenic dyskinesia, episodic ataxia, epileptic syndromes such as benign familial infantile seizures, and hemiplegic migraine^1^. The majority of the mutations described until now are predicted to cause a loss of function of the protein. However, no clear genotype–phenotype correlation has been identified^1,2^. Most of the mutations are found in heterozygosity. Few patients with biallelic mutations have been reported to exhibit very severe manifestations with developmental delay and intellectual disability, thus suggesting a gene-dosage effect and an implication of the protein in neurodevelopmental processes^3^.

The *PRRT2* gene encodes for a neuronal protein that participates in Ca^2+^-dependent synaptic vesicle fusion and neurotransmitter release by interacting with components of the soluble N-ethylmaleimide-sensitive factor attachment protein receptors (SNARE) complex, namely synaptosomal nerve-associated protein 25 (SNAP-25), vesicle-associated membrane protein 1 (VAMP1), syntaxin 1B (STX1B) and with the Ca^2+^ sensors synaptotagmins 1 and 2 (SYT1/2)^4^. Moreover, it was recently described that PRRT2 regulates the membrane exposure and recovery from inactivation of the voltage-gated sodium channels 1.2 and 1.6 subtypes responsible for the generation of action potentials in the axonal initial segment^5^. These results highlight a fundamental role of PRRT2 in stabilizing neuronal excitability^6^, in-line with the hyperexcitability and paroxysmal manifestations observed in patients and mouse models in which PRRT2 is mutated^7,8^.

It has also been reported that the silencing of PRRT2 during mouse brain development delays the migration of postmitotic neurons from the ventricular zone to the cortical plate^9^, and that PRRT2 interferes with the migration of glioblastoma cell lines^10^. Furthermore, PRRT2 knockdown in neurons in culture during synaptogenesis strongly disrupts synaptic density^4^. This evidence suggests that, in addition to its role in the regulation of neuronal transmission, PRRT2 might have an unexpected role in neuronal cell architecture and, at the same time, in the control of neuronal motility and synapse formation.

The driving force shared between neuronal motility and synaptic morphogenesis is the remodeling of the actin cytoskeleton^11^. In neurons, actin is the major cytoskeletal component at both the pre and postsynaptic terminals. The control of actin polymerization underlies the morphological rearrangements occurring during synaptogenesis and synaptic plasticity phenomena^12^. The intimate relationship between the actin cytoskeleton and synaptic activity implies that some mediators perceive the activation state of the neurons and convey it to actin-related signaling pathways.

Here we demonstrate that PRRT2-depleted hippocampal neurons display an alteration of actin polymerization and turnover within synapses due to an aberrant function of cofilin, an actin-binding protein promoting cytoskeleton dynamics. These actin-related disturbances are responsible for the structural defects found in PRRT2-silenced neurons, as they are rescued by normalizing cofilin activity. Together, we identified a novel function of PRRT2 in the regulation of actin cytoskeleton that may play a role in the alterations of structural and functional connectivity resulting from PRRT2 mutations.

## Materials and methods

### Animals

Wild-type (WT) C57BL6N mice were obtained from Charles River (Calco, Italy). Mice were housed under constant temperature (22 ± 1 °C) and humidity (50%) conditions with a 12 h light/dark cycle and were provided with food and water ad libitum. All experiments involving animals followed protocols in accordance with the guidelines established by the European Communities Council (Directive 2010/63/EU of March 4, 2014) and were approved by the Institutional Animal Care and Use Committee (IACUC, permission number 796) of the San Raffaele Scientific Institute and the Italian Ministry of Health. All efforts were made to obey the 3Rs principle and to minimize animal suffering.

### Plasmids and lentiviral vectors

The preparation of the PRRT2-EGFP construct has been previously described^4^. The mouse *Prrt2* cDNA coding sequence from 799 to 1039 bp lacking the N-terminal domain (∆N-*Prrt2*) was subcloned into the p-EGFP-N3 vector (BD Bioscience, Difco, Franklin Lakes, NJ, USA). The human cofilin (cofilin WT) plasmid was a kind gift of Dr. Alessandro Vacchini^13^. Cofilin S3A and S3E mutants were generated by site-directed mutagenesis (QuickChange Lightning Mutagenesis Kit, Agilent Technologies) using the following primers: human cofilin S3A_for GGATCCATGGCCGCCGGTGTGGCTG, human cofilin S3A_rev CAGCCACACCGGCGGCCATGGATCC, human cofilin S3E_for AGGGGGATCCATGGCCGAAGGTGTGGCTGTCTCTG, human cofilin S3E_rev CAGAGACAGCCACACCTTCGGCCATGGATCCCCCT. Human Cofilin WT, S3A and S3E sequences fused to a mCherry tag were inserted in a p743.pCCLsin.PTT.hPGK.GFP.Wpre_mut_AMP lentiviral vector, provided by Prof. Luigi Naldini (TIGET, IRCCS San Raffaele Scientific Institute, Milan, Italy). PRRT2 silencing and control (scramble) shRNA sequences inserted into a pLKO.1-CMV-mCherry bicistronic lentiviral vector carrying an mCherry reporter were previously generated and characterized^4^. A pLKO.1-CMV-mTurquoise version of the shRNAs was also produced, by amplifying the mTurquoise sequence from an FU-PSD95-mTurq-Wm vector (kindly provided by Prof. Noam Zivì, Technion, Haifa, Israel^14^) and substituting it to the mCherry. An Sh-resistant version of PRRT2 (rPRRT2) fused to the mCherry reporter and inserted in the p743.pCCLsin.PTT.hPGK.GFP.Wpre_mut_AMP lentiviral vector was previously generated and characterized^4^. Viral stocks production and titration via FACS sorting was performed as previously described^15,16^. All primers were purchased from Sigma–Aldrich (Sigma, Milan, Italy) and all restriction enzymes were from New England Biolabs (NEB, Ipswich, MA, USA).

### Cell culture procedures

HeLa and NIH 3T3 cells were grown in Dulbecco’s Modified Eagle’s Medium (DMEM; Gibco-ThermoFisher, Waltham, Massachusetts, USA #21969-035) supplemented with 10% fetal bovine serum (FBS; Euroclone, Pero, Italy #ECS0180LH), 1% L-glutamine (Sigma #G7513) and 1% penicillin/streptomycin (P/S; Sigma #P0781). HEK293T cells were grown in Iscove’s Modified Dulbecco’s Medium (IMDM; Sigma #13390) supplemented with 10% FBS, 1% glutamine and 1% P/S. All cell lines were kept at 37 °C in 5% CO_2_ humidified atmosphere.

Primary neuronal cultures were prepared from the hippocampi of embryonic day 17.5 embryos from WT mice of either sex, as previously described^17,18^. For low-density cultures, neurons were plated on poly-L-lysine (0.2 mg/mL; Sigma #P2636)-coated 24 mm glass coverslips at a density of 120,000 cells per coverslip, and maintained as a sandwich co-culture with astroglia in Modified Eagle’s Medium (MEM Invitrogen, Carlsbad, CA, USA) supplemented with 1% N2 supplement (Gibco #1502-048), 2 mM L-glutamine, 1 mM sodium pyruvate (Sigma #P2256) and 4 mM glucose at 37 °C in 5% CO_2_ humidified atmosphere^19^. For high density cultures, glass coverslips were coated with 0.2 mg/mL poly-L-lysine, washed and air-dried. Hippocampal cells were diluted in Neurobasal medium (Gibco #21103-049) supplemented with 10% FBS, 2% B27 (Gibco #17504-044), 1% P/S and 1% L-glutamax (Invitrogen #35050-038) in order to have 80,000 cells in 200 µL for each coverslip. Cells were then plated as a drop in the middle of the coverslip. After 4–5 h, serum-free Neurobasal medium was added.

Transient transfection of cell lines was performed with Lipofectamine-2000 (Invitrogen #11668-019), following manufacturer’s instruction. Transient transfection of primary neurons was performed with Lipofectamine-2000 at 4 days in vitro (DIV), in a clean dish containing a half fresh and half conditioned complete Neurobasal medium. Neurons were incubated with the transfection mixture for 1 h and then returned to the original dishes. For lentiviral transduction, coverslips were placed in a clean dish containing a mixture of fresh and conditioned medium and were incubated overnight in the presence of viral stocks at a multiplicity of infection (MOI) of 2. For rescue experiments, neurons were co-transduced with lentiviruses expressing shRNA and lentiviruses expressing the Sh-resistant PRRT2 (2 MOI + 2 MOI). After transduction, neurons were returned to the original dishes and maintained in culture until analysis. For biochemical experiments, lentiviruses were added directly to the cell medium with no subsequent medium change. The MOI of 2 was selected upon optimization by western blot analysis in order to estimate the toxicity of the transduction and to obtain efficient downregulation of the PRRT2 protein. If not otherwise specified, neurons were infected at 7 DIV and analysed after one week (14 DIV).

### Wound-healing assay

NIH 3T3 cells were transfected with vectors expressing PRRT2-EGFP or EGFP as a control. The transfection efficiency, calculated as EGFP-positive cells on the total number of cells, was around 20–30%. After 72 h cells were split into 6-well plates at 50–70% confluence. After 24 h, when cells reached 100% confluence, they were starved in 0.5% FBS medium for 8 h. The confluent cell monolayer was scratched using a micropipette tip. After 30 min in the incubator, cells were monitored by phase-contrast microscopy at ×10 magnification every 30 min for 16 h using an Axiovert GFP-imaging microscope (Zeiss, Oberkochen, Germany). Six independent regions of the scratch were considered for each condition. The migration distance was measured from the analysis of the movies with the MRI-Wound Healing Tool of ImageJ. To quantify cell migration, the area of the initial wound was compared with the area of the healing wound at various time points after the scratch, where % Healed = [(Area of original wound − Area of wound during healing)/Area of original wound]×100^20^. The cumulative value of the empty spaces among cells at all single frames was measured using the same ImageJ plug-in.

### Cell aggregation assay

HEK293T cells were transfected with vectors expressing PRRT2-EGFP, the N-terminal deletion mutant of PRRT2 (ΔN-PRRT2-EGFP) or EGFP as controls. After 24 h, cells were detached with PBS containing 2 mM EDTA, centrifuged at 1 200 × *g* for 5 min and resuspended in 1X Hank’s Balanced Salt Solution (HBSS; Gibco #14170-088) supplemented with 2 mM CaCl_2_ and 1 mM MgCl_2_. 2.0 × 10^6^ cells in a total volume of 500 μL were incubated at RT under rotation. After 30 min 100,000 cells (25 μL) from each suspension were plated onto poly-L-lysine-coated coverslips for 10 min, fixed and processed for immunofluorescence as described below with an antibody directed against β-catenin to delimit the cell area and with Hoechst 33342 (Cell Signaling, Danvers, MA, USA #4082 S) for nuclear staining. For each experiment, three coverslips were plated per condition and 10 images per coverslip were taken from random fields at ×63 magnification with a Zeiss Axio Vision microscope. For each image, the total number of cells, the number of clusters (>2 cells) and the number of cells per cluster were quantified.

### G/F-actin ratio assay

Hippocampal neurons were infected at 7 DIV with MOI 2 of Scramble, ShPRRT2 or Sh+rPRRT2 lentiviruses. Noninfected cells were used as positive control and treated at 11 DIV with phalloidin, to stabilize F-actin filaments for 10 min at 37 °C. Separation of F- to G-actin was performed with the “G-actin/F-actin In Vivo Assay Biochem” kit (Cytoskeleton Inc, Denver, CO, USA #BK037) following the manufacturer’s instructions. Briefly, cells were incubated with warm LAS2 buffer composed by a lysis buffer, a stabilizer of F-actin, 100 mM ATP and protease inhibitors. Cells were scraped, collected and mechanically homogenized. The lysate was then incubated for 10 min at 37 °C. Part of the lysate was resuspended in Laemmlii buffer (final concentrations: 20 mM Tris-HCl pH 6.8, 2 mM EDTA, 2% SDS, 10% glycerol, 2% β-mercaptoethanol and 0.01% bromophenol blue) and stored at −80 °C until use for western blotting as “Input” fraction. The remaining part of cell lysate was centrifuged at 350 × *g* for 5 min at RT. The pellet was discarded, while the supernatant was centrifuged at 100,000 × *g* for 1 h at 37 °C (TLX ultra, 120.2 rotor). The supernatant obtained from the ultracentrifugation contains G-actin monomers. The pellet, enriched in F-actin, was resuspended in a depolymerising buffer provided in the kit and incubated on ice for 1 h to allow F-actin depolymerization. Both G- and F-actin fractions were resuspended in Laemmli buffer and stored at −80 °C until use. Western Blot analysis was performed with anti-actin and anti-PRRT2 antibodies, as described in the supplementary procedures.

### Cell labelling protocols and image acquisition

Immunofluorescence experiments were performed as previously described^21^. Briefly, cells were rinsed once with phosphate buffer saline (PBS; Gibco #14200-056) for cells lines or Krebs-Ringer’s solution (KRH)-EGTA (in mM: 130 NaCl, 5 KCl, 1.2 KH_2_PO_4_, 1.2 MgSO_4_, 2 MgCl_2_, 2 EGTA, 25 HEPES and 6 glucose, pH 7.4) for neurons. Cells were fixed for 15 min with 4% paraformaldehyde (Sigma #441244), 4% sucrose in 120 mM sodium phosphate buffer, pH 7.4, supplemented with 2 mM EGTA. Coverslips were rinsed three times with PBS and then incubated at RT for 2 h or overnight at 4 °C in a humidified chamber with the primary antibodies appropriately diluted in goat serum dilution buffer (GSDB; 15% goat serum, 450 mM NaCl, 0.3% Triton X-100, and 20 mM sodium phosphate buffer, pH 7.4). Specimens were then washed three times with PBS and incubated with the appropriate secondary antibodies at RT for 1 h. After three washes with PBS, coverslips were mounted with Dako fluorescence mounting medium (Dako, Carpinteria, CA, USA #S3023). Labelling of cofilin-actin rods was performed as described in ref. ^22^. Briefly, neurons were rinsed once with KRH-EGTA, fixed 10 min with 4% paraformaldehyde, 4% sucrose in 120 mM sodium phosphate buffer, pH 7.4, supplemented with 2 mM EGTA, and permeabilized with methanol at −20 °C for 10 min followed by 20 min in 1% Triton X-100, 4% horse serum (HS) in PBS. Primary and secondary antibodies were diluted in PBS containing 1% HS. Alexa Fluor 488-conjugated phalloidin (ThermoFisher, Waltham, MA, USA #A12379) was incubated together with the secondary antibodies (diluted 1:300) when indicated. The following primary antibodies were used: β-catenin (Stressgen #ALX-804-260-C100 Mouse) 1:150, Paxillin (Sigma #051309 Rabbit) 1:100, pTyr^397^FAK (ThermoFisher #44-6246 Rabbit) 1:200, TuJ1 (Biolegend #801201 Mouse) 1:1000, Bassoon (Stressgen #ADI-VAM-PS003-F Mouse) 1:150, Homer1 (Synaptic Systems, Goettingen, Germany #160 003 Rabbit) 1:200, Cofilin (Cell Signaling #5175 Rabbit) 1:200. The following secondary antibodies were used: FITC anti-mouse (Jackson ImmunoResearch #115-095-205) 1:50, TRITC anti-mouse (Jackson ImmunoResearch, West Grove, PA, USA #715-025-151) 1:50, TRITC anti-rabbit (Jackson ImmunoResearch #715-025-152) 1:50. When indicated, nuclei staining was performed by incubating coverslips with the Hoechst 33342 dye (ThermoFisher) diluted 1:10 000 in PBS for 5 min during the last round of washes. Epifluorescence images were acquired with either Zeiss Axio Observer Z1 equipped with an ImagEM X2 EM-CCD camera or with Zeiss Axio Vision equipped with an AxioCam MRm camera, with ×63 objectives. For the analysis of cofilin-actin rods, images were blindly acquired and analyzed.

### Analysis of the intensity of phalloidin staining at synaptic puncta

Neurons were infected at 7 DIV with lentiviruses and processed at 14 DIV for immunofluorescence. Bassoon, Alexa Fluor 488-phalloidin dye and Hoechst 33342 staining were used in order to stain synapses, F-actin and nuclei, respectively. Thirty neurons for each condition were acquired with Zeiss Axio Vision with ×63 objective. Images were acquired at 16-bit resolution to increase the grayscale range of intensities. 0.1–1 μm^2^ brighter points, representing synaptic puncta, were selected on the Bassoon channel with the Analyze Particles tool from ImageJ and identified as ROIs that were resized to a defined round area (7 × 7 pixels). The mean phalloidin fluorescence intensity in the ROIs was measured. All values were normalized to the mean of the scramble values for each experiment. Data were expressed as the frequency distribution of the percentage of puncta on the normalized fluorescence intensity. The parameters describing the distribution, namely median, coefficient of variation (describing the dispersion of data) and coefficient of skewness (describing the symmetry of the distribution) were extrapolated and compared among groups.

### Synaptic density analysis

Low-density hippocampal neurons were infected at 7 DIV with lentiviral vectors and processed at 14 DIV for immunofluorescence as described previously. A presynaptic (Bassoon) and a postsynaptic (Homer1) markers were used to identify synaptic contacts. Images were acquired with Zeiss Axio Observer Z1 with ×63 objective. Five dendrites for each neuron, from at least 10 neurons for each sample were analysed, by isolating 30-μm segments starting from the soma. A synapse was defined as a colocalization point (>50% overlap) between Bassoon and Homer1 puncta identified with the “Colocalization highlighter” plug-in of ImageJ. 0.1–1 μm^2^ puncta were then counted with the Analyze Particles tool of ImageJ.

### Spine density and morphometric analysis

High density hippocampal neurons were transfected at 4 DIV with 1 μg of p-EGFP vector and infected at 7 DIV with the appropriate lentiviral vectors. Neurons were processed for immunofluorescence at 14 DIV. Confocal images of hippocampal neurons were acquired using Leica SP8 SMD laser scanning confocal, using a 63X objective applying a 2× zoom. Each image derives from a Z series projection of 5–10 stacks, taken at 0.3 μm depth intervals with a 1024 × 1024 pixel resolution. Morphometric spine measurements were performed using the NeuronStudio software considering the following classification: mushroom (length ≤ 1.2 μm, width ≥ 0.5 μm); filopodia (length > 1.2 μm, width < 0.5 μm); stubby (length ≤ 0.5 μm, width ≤ 0.5 μm)^23^.

### Fluorescence recovery after photobleaching (FRAP) assay

Hippocampal neurons were transfected with 0.5 μg of EGFP-actin at 4 DIV and transduced at 7 DIV with either Scramble-mTurquoise, ShPRRT2-mTurquoise or Sh+rPRRT2-mCherry lentiviruses. FRAP was performed at 14 DIV using Leica TSC SP8 confocal microscopy equipped with a stage incubator for temperature (37 °C), CO_2_ (5%) and humidity control (Oko-Lab #H301ECLGBL). During the acquisition, neurons were maintained in KRH solution (in mM: 130 NaCl, 5 KCl, 1.2 KH_2_PO_4_, 1.2 MgSO_4_, 2 CaCl_2_, 25 HEPES and 6 glucose, pH 7.4, 315 mOsm). Images of 512 × 512 pixels were acquired with a ×63/1.4 NA oil immersion objective, with a 6× digital zoom, using a 488 nm Argon laser at 2% power, 800 V Gain, 400 Hz scanning speed and 2.5 AU pinhole. Fifteen prebleaching images were acquired at 0.8 Hz. Bleaching was performed using a 488 nm Argon laser at 70% power with 10 pulses on the region of interest (circular ROI, 1.3 μm diameter). After bleaching, 30 images were acquired at 0.8 Hz, followed by 30 images at 0.5 Hz and 30 images at 0.1 Hz to monitor the fluorescence recovery over time. The average pixel intensity was measured in the ROIs at each timepoint, and data were normalized to the average prebleaching intensity. *f* mobile is the fraction of fluorescence corresponding to diffusing monomers and is identified as the fluorescence intensity measured in the first postbleaching frame. *f* stable corresponds to the recovery of more stable structures and is measured as 1–Ymax, where Ymax is the intensity measured in the last postbleaching frame. The dynamic fraction describes the incorporation of monomers in polymers and is calculated as (*f* stable − *f* mobile). The recovery curve was fitted to the following equation: *f* = *f* mobile + *f* dynamic × [1 − exp(−t/τ)]. We calculated the turnover rate of actin filaments as 1/τ, where τ is the timepoint at which the dynamic component reached half the value of full recovery^24,25^_._

### Electrophysiological recordings

Whole-cell patch-clamp recordings were performed using pipettes prepared from thin borosilicate glass (Hilgenberg, Mansfield, Germany) pulled (Narishge PP-83) and fire-polished to a final resistance of 2–4 MΩ when loaded with a standard intracellular solution. This contained (in mM): 126 K-gluconate, 4 NaCl, 1 MgSO_4_, 0.02 CaCl_2_, 0.1 BAPTA, 15 glucose, 5 HEPES, 3 ATP, 0.1 GTP (pH 7.2 with KOH). Neurons were maintained in an extracellular solution with the following composition (in mM): 140 NaCl, 2 CaCl_2_, 1 MgCl_2_, 4 KCl, 10 glucose, 10 HEPES (pH 7.3 with NaOH). Currents were recorded using a double EPC-10 amplifier (HEKA Electronic, Lambrecht, Germany). Miniature excitatory postsynaptic currents (mEPSCs) were collected from low-density hippocampal neurons voltage-clamped at −70 mV, in the presence of tetrodotoxin (TTX, 1 μm, Tocris, Minneapolis, USA #1078/1) to block spontaneous action potentials (APs). Moreover, extracellular solution was supplemented with D-(-)-2-Amino-5-phosphonopentanoic acid (D-AP5; 50 μM, Tocris #0106), Bicuculline methiodide (30 μm, Tocris #2503/10) and CGP 55845 hydrochloride (10 μM, Tocris #1248/10), to block NMDA, GABA_A_ and GABA_B_ receptors, respectively. Low-density hippocampal cultures were infected at 7 DIV with either Scramble or ShPRRT2 lentiviral vectors, in combination with S3E- or S3A-cofilin lentivirus. mEPSCs were registered at 11–14 DIV at RT (22–24 °C). Currents were acquired at 10–20 kHz sample frequency and filtered at half the acquisition rate with an 8-pole low-pass Bessel filter. Recordings with leak currents >100 pA or series resistance >20 MΩ were discarded. Data acquisition was performed using PatchMaster (HEKA Elektronic). mEPSC analysis was performed by using Minianalysis (Synaptosoft Inc., Leonia, NJ, USA). The amplitude and frequency of mEPSCs were calculated using a peak detector function with appropriate threshold amplitude and threshold area.

### Statistical analysis

Data were analysed using Microsoft Excel and GraphPad Prism 8.0 (La Jolla, CA, USA). Normal distribution of data was evaluated using D’Agostino–Pearson normality test. In case of normally distributed data, statistical comparisons were performed with either the two-sided Student’s *t*-test or one-way ANOVA followed by Bonferroni’s multiple comparison test. When the variance between groups was different, Welch’s correction was applied and stated in the figure legend. When data distribution was not normal, either the Mann–Whitney’s *U* test or Kruskal–Wallis analysis of variance followed by Dunn’s multiple comparison test was used. Sample size for each experiment was calculated using G Power (ver. 3.1) software and based on effect sizes calculated from our preliminary or based previously published data (appropriately referred to in the References), with a power of 0.8 at the alpha = 0.05 level.

## Results

### Ectopically expressed PRRT2 delays wound healing and increases cell-to-cell adhesion

**Fig. 1:**
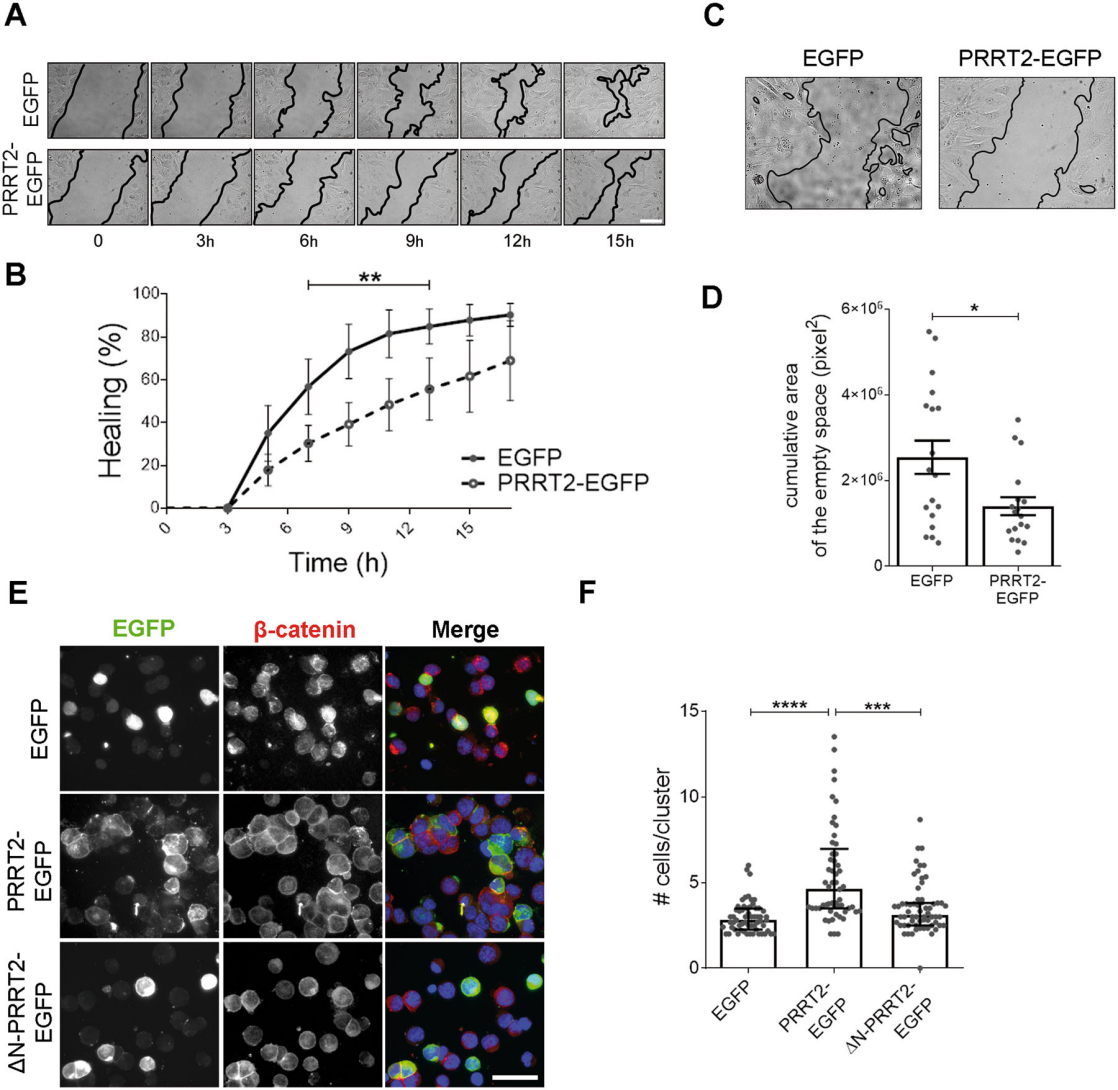
Expression of PRRT2 in heterologous cells reduces cell motility and promotes cell aggregation. **A** Phase-contrast images of NIH 3T3 fibroblasts expressing either EGFP (control) or PRRT2-EGFP taken from timepoint 0 (30 min after scratching) and at the indicated time intervals (h) and showing the wound closure progression. Leading edges are labelled in black for easier visualization. Scale bar: 20 μm. **B** Quantification of the wounded area invaded at different time points, expressed as percentage of healing at time 0. % Healing: 7 h EGFP = 56.7 ± 12.9, PRRT2-EGFP = 30.3 ± 8.4; 9 h EGFP = 73.2 ± 12.7, PRRT2-EGFP = 39.2 ± 10.1; 11 h EGFP = 81.4 ± 11.2, PRRT2-EGFP = 48.3 ± 12.2; 13 h EGFP = 84.8 ± 8.1, PRRT2-EGFP = 55.6 ± 14.5; *p* < 0.01. Results represent the mean ± SEM of *n* = 3 independent experiments, with six movies/group in each experiment. Two-way ANOVA/Bonferroni’s tests; **p < 0.01. **C** Phase-contrast images denoting the empty area among cells treated as described in **A**. **D** Quantification of the area among cells expressed as cumulative area of empty spaces among cells measured in all the frames. Sum of the empty area (pixel^2^) EGFP = 2,539,468 ± 387,867, PRRT2-EGFP = 1,395,844 ± 209,285; *p* < 0.05. Data are expressed as means ± SEM of *n* = 18 movies from three independent experiments. Student’s *t*-test + Welch-correction; *p < 0.05. **E** Fluorescence images of HEK293T cells transfected with plasmids coding for either EGFP, PRRT2-EGFP or ∆N-PRRT2-EGFP. Cells were incubated in adhesion favouring conditions. At the end of the assay, cells were fixed and processed for immunofluorescence with β-catenin antibody (red) to label cell edges and Hoechst 33342 to label nuclei (blue). Scale bar: 50 μm. **F** Quantification of the cell number/cluster from the experiments shown in **E**. Median n. of cells/cluster: EGFP = 2.8, PRRT2-EGFP = 4.6, ∆N-PRRT2-EGFP = 3.1; *p* < 0.0001 EGFP vs PRRT2-EGFP, *p* < 0.001 PRRT2-EGFP vs ∆N-PRRT2-EGFP. Results are expressed as median ± interquartile range from *n* = total number of fields from three independent experiments carried out in triplicate (total number of fields: EGFP = 60, PRRT2-EGFP = 57, ΔN-PRRT2-EGFP = 62). Kruskal–Wallis/Dunn’s tests; ***p < 0.001, ****p < 0.0001.

Starting from literature evidence reporting an effect of PRRT2 expression in the migration of neuronal precursors and tumor cells^9,10^, we performed a wound-healing assay in NIH 3T3 fibroblasts, to gain insight into the mechanisms underlying the effect of PRRT2 on cell motility. Cells were transfected with either EGFP or PRRT2-EGFP and, after the production of a scratch in the cell monolayer, the healing was followed over time with time-lapse imaging. PRRT2-EGFP expression reduced the speed of wound closure as compared to the control condition (Fig. 1A, B). Moreover, we observed that the migration front was more compact in cells expressing PRRT2-EGFP, as shown by the reduction of the empty space among cells (Fig. 1C, D). This observation points to a decreased cell motility, possibly due to an increased cell-to-cell adhesion in the presence of PRRT2. To further verify the activity of PRRT2 on cell adhesion, we performed a cell aggregation assay in HEK293T cells expressing either EGFP, PRRT2-EGFP or a truncated form of PRRT2, which lacks the intracellular N-terminal domain (ΔN-PRRT2-EGFP). The expression of PRRT2-EGFP, but not of ΔN-PRRT2-EGFP, stimulated the formation of cell clusters composed by a higher number of cells as compared to control (Fig. 1E, F).

### The ectopic expression of PRRT2 in cell lines stabilizes cell-to-ECM adhesion and filopodia

**Fig. 2:**
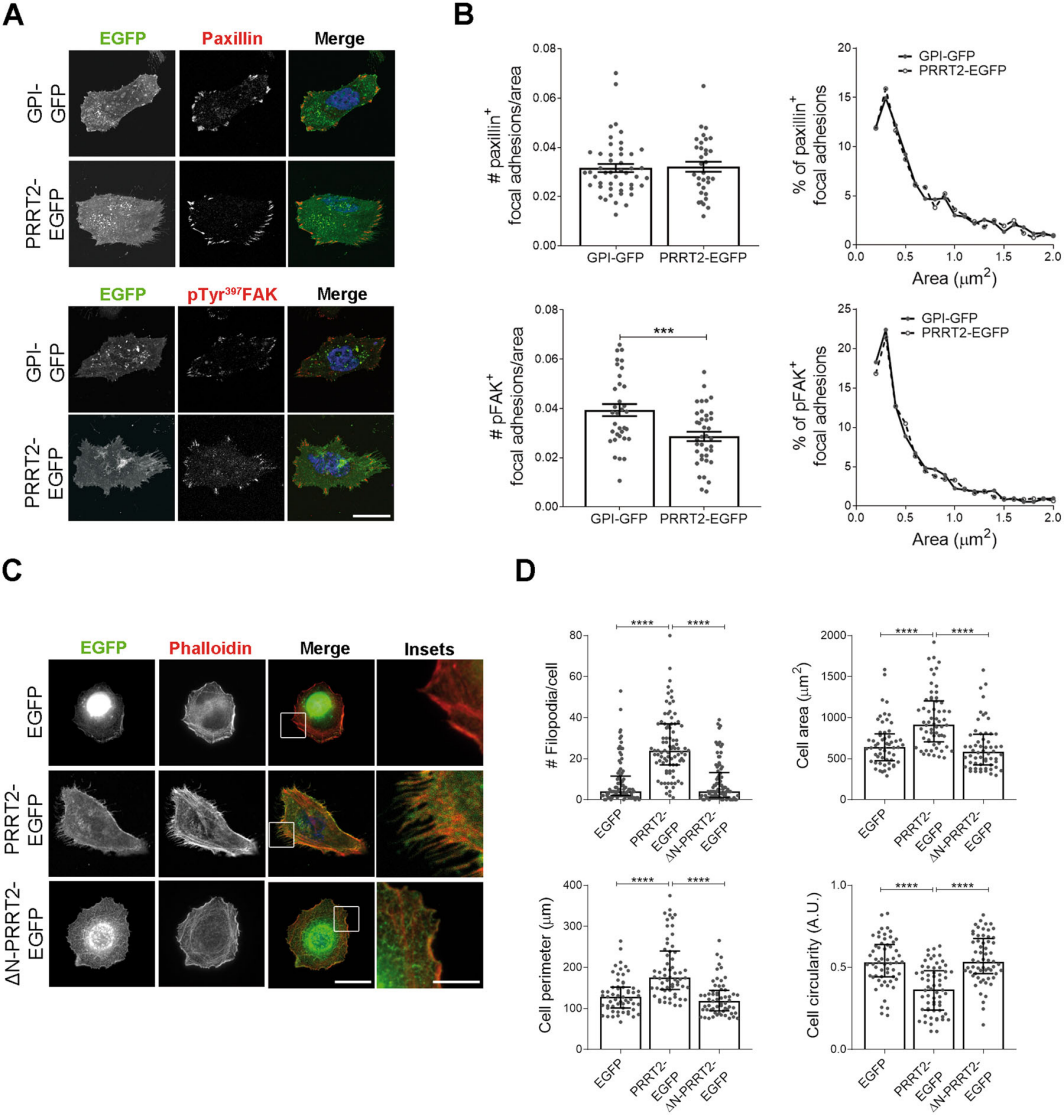
Expression of PRRT2 in heterologous cells promotes the stabilization of focal adhesions and filopodia. **A** Confocal images of HeLa cells overexpressing PRRT2-EGFP or GPI-GFP as control. Cells were stained for paxillin (upper) or pTyr^397^FAK (lower) to mark focal adhesions (red). Scale bar: 20 μm. **B** Quantitative analysis of the number of focal adhesions/cell area (left) and of their size frequency distribution (right) for paxillin (upper panels) or pFAK (lower panels) staining, respectively. Mean paxillin^+^ focal adhesions/cell area ± SEM: GPI-GFP = 0.038 ± 0.005, PRRT2-EGFP = 0.041 ± 0.004. Mean pFAK^+^ focal adhesions/cell area ± SEM: GPI-GFP = 0.039 ± 0.002, PRRT2-EGFP = 0.028 ± 0.002; p < 0.001. Median area of paxillin^+^ focal adhesions (μm^2^): GPI-GFP = 0.574, PRRT2-EGFP = 0.583; median area of pFAK^+^ focal adhesions (μm^2^): GPI-GFP = 0.416, PRRT2-EGFP = 0.444. Data are means ± SEM of *n* = total number of cells from three independent experiments (GPI-GFP paxillin = 48, PRRT2-EGFP paxillin = 33, GPI-GFP pFAK = 35, PRRT2-EGFP pFAK = 38). Student’s *t*-test, ***p < 0.001. **C** Representative images of HeLa cells transfected with plasmids coding for EGFP (control), PRRT2-EGFP or ∆N-PRRT2-EGFP. Cells were fixed and incubated with 568-Phalloidin dye (red) to stain F-actin and with Hoechst 33342 solution (blue) to stain nuclei. Scale bar: 20 μm (insets: 5 μm). **D** Analysis of the number of filopodia and of the area, perimeter and circularity of cells treated as described in **C**. Only protrusions extending more than 2 μm from the cell edge were considered. Median # filopodia/cell: EGFP = 4, PRRT2-EGFP = 24, ∆N-PRRT2-EGFP = 4; *p* < 0.0001 EGFP vs PRRT2-EGFP, *p* < 0.0001 PRRT2-EGFP vs ∆N-PRRT2-EGFP. Median cell area (μm^2^): EGFP = 639.7, PRRT2-EGFP = 915.6, ∆N-PRRT^2^-EGFP = 582.1; *p* < 0.0001 EGFP vs PRRT2-EGFP; *p* < 0.0001 PRRT2-EGFP vs ∆N-PRRT2-EGFP. Median cell perimeter (μm): EGFP = 128.0, PRRT2-EGFP = 175.1, ∆N-PRRT2-EGFP = 118.8; *p* < 0.0001 EGFP vs PRRT2-EGFP, *p* < 0.0001 PRRT2-EGFP vs ∆N-PRRT2-EGFP. Median cell circularity (A.U.): EGFP = 0.53, PRRT2-EGFP = 0.37, ∆N-PRRT2-EGFP = 0.54: *p* < 0.0001 EGFP vs PRRT2-EGFP; *p* < 0.0001 PRRT2-EGFP vs ∆N-PRRT2-EGFP. Data in the figure are shown as median ± interquartile range of *n* = 90 cells from 3 independent experiments. Kruskal–Wallis/Dunn’s tests; ****p < 0.0001.

To verify the involvement of PRRT2 in regulating cellular adhesion, we measured the number and area of focal adhesions in HeLa cells expressing PRRT2 compared to control cells. Focal adhesions were immunostained for paxillin, an adaptor protein involved in signal transduction at focal adhesions, and for focal adhesion kinase (FAK) phosphorylated on tyrosine 397 residue, which labels dynamic focal adhesions that initiated their turnover^26^. We did not find any significant alteration in the number of paxillin-positive focal adhesions in PRRT2-EGFP expressing cells as compared to control. In contrast, the number of pTyr^397^FAK-positive focal adhesions was reduced upon expression of the EGFP-tagged PRRT2, suggesting that the PRRT2 ectopic expression alters focal adhesion retrieval, which is known to influence the cellular adhesive ability. No significant effect was found on the size of focal adhesions (Fig. 2A, B).

Filopodia are cell appendices that probe the environment to guide cellular migration. They are stabilized by adhesion to the extracellular matrix (ECM) through focal adhesions, which tightly connect the ECM to the actin cytoskeleton^27,28^. We observed that the expression of PRRT2-EGFP in HeLa cells (Fig. 2C, D), as well as in other cells lines (Supplementary Fig. 1), induced the formation of actin-rich filopodia stained by phalloidin, a toxin that binds and stabilizes F-actin. Furthermore, cell shape, which is largely dependent on actin cytoskeleton, was modified by PRRT2 ectopic expression. Indeed, the area and the perimeter of the cells increased upon expression of PRRT2-EGFP, while their circularity decreased, indicating a more complex cell shape. Interestingly, all these effects were ablated in cells expressing ∆N-PRRT2-EGFP. Since filopodia initiation is mainly regulated by the activity of the small G protein Cdc42^29^, we compared the activation state of the protein between EGFP or PRRT2-EGFP overexpressing cells, but no significant differences were found (Supplementary Fig. 2).

### PRRT2 silencing in hippocampal neurons in culture decreases the F-actin concentration at synapses

**Fig. 3:**
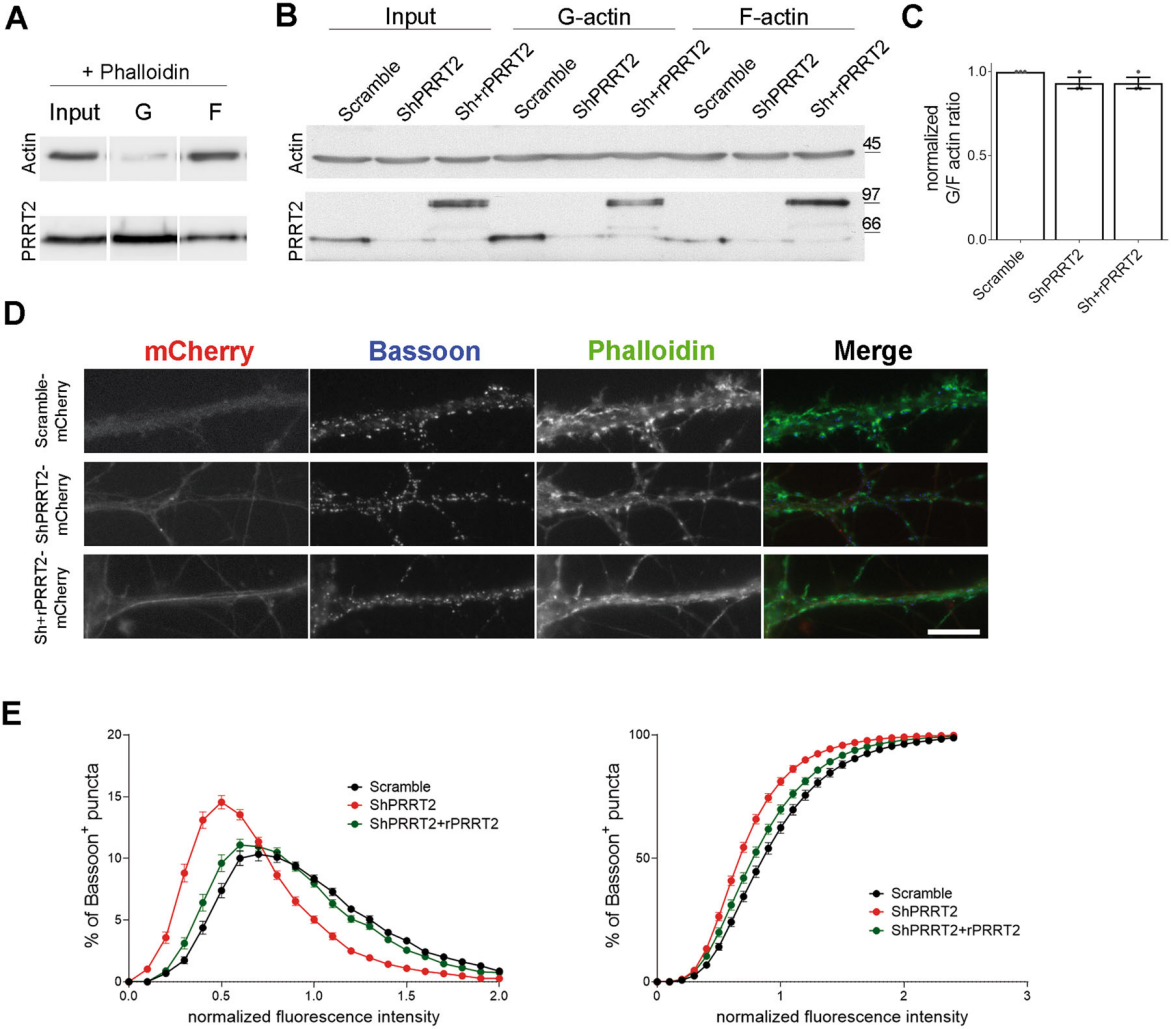
Knockdown of PRRT2 in hippocampal neurons disrupts the F-actin cytoskeleton. Representative immunoblot of PRRT2 and actin protein levels in **A** noninfected hippocampal neurons treated at 11 DIV with phalloidin (positive control) or **B** neurons infected at 7 DIV with mCherry-tagged either Scramble, ShPRRT2 or Sh+rPRRT2 lentiviruses and processed for G-/F-actin separation at 11 DIV. For each sample, equal volumes of pellet (F-actin fraction) and supernatant (G-actin fraction) were loaded. As expected, in the positive control (**A**) the F-actin fraction is predominant as compared to the G-actin pool. **C** Densitometric analysis of protein levels expressed ad G/F-actin ratio. Data are normalized on the mean of Scramble samples. Scramble = 1, ShPRRT2 = 0.93 ± 0.06, Sh+rPRRT2 = 0.93 ± 0.06. G/F-actin ratio is expressed as mean ± SEM of *n* = 3 independent experiments. One-way ANOVA/Bonferroni’s tests. **D** Representative mCherry-tagged hippocampal neurons infected at 7 DIV with either Scramble, ShPRRT2 or Sh+rPRRT2 lentiviruses. At 14 DIV neurons were fixed and stained by immunofluorescence with a presynaptic marker (Bassoon, blue) and with 488-phallodin (green) to mark F-actin. 0.1–1 μm^2^ brighter points on the Bassoon channel were automatically identified by the ImageJ software, and the average intensity of phalloidin in the ROIs of defined size (7 × 7pixel) was measured. Scale bar: 20 μm. **E** Quantitative analysis of the frequency distribution of the intensity of the phalloidin signal in Bassoon-positive puncta shown as frequency (left panel) or cumulative (right panel) distribution. Data are expressed as distributions obtained from *n* = 90 fields for each group from 3 independent experiments normalized on the mean value of the Scramble group. Friedman/Dunn’s tests *p* < 0.05 Scramble vs ShPRRT2. Descriptive curve parameters Scramble: median = 1.996 ± 0.114, coefficient of variation (cov) = 114.7 ± 2.7, coefficient of skewness (cos) = 0.96 ± 0.05; ShPRRT2: median = 1.258 ± 0.115 (*p* < 0.001), cov = 137.7 ± 3.2 (*p* < 0.001), cos = 1.34 ± 0.05 (*p* < 0.001); Sh+rPRRT2: median = 1.841 ± 0.108 (*p* < 0.001 vs ShPRRT2), cov = 119.9 ± 2.7 (*p* < 0.001 vs ShPRRT2), cos = 1.08 ± 0.05 (p < 0.01 vs ShPRRT2); Kruskal–Wallis/Dunn’s tests.

Given the information gathered in cell lines, we evaluated in hippocampal neurons the effect of manipulation of PRRT2 levels on actin dynamics. We first studied whether the loss of PRRT2 causes a general unbalance between monomeric (G-) and filamentous (F-) actin pools. To this aim, 7 DIV neurons were infected with a lentiviral vector containing either a short hairpin RNA to induce the silencing of PRRT2 (ShPRRT2) or an unrelated shRNA (Scramble), along with a mCherry fluorescent reporter to identify transduced neurons. To verify the specificity of the phenotypes, some samples were co-infected with a lentivirus coding for a mCherry-tagged Sh-resistant PRRT2 (rPRRT2). Infection of hippocampal neurons at MOI 2 with the appropriate lentiviral vectors resulted in an effective silencing of PRRT2 and in the expression of the shRNA-resistant version of PRRT2 (Supplementary Fig. 3). At 11 DIV, neurons were subjected to a G/F-actin ratio assay, which is based on the use of a lysis buffer that stabilizes actin filaments so that upon ultracentrifugation F-actin is found in the pellet and G-actin remains soluble in the supernatant (Fig. 3A). Neurons silenced for PRRT2 did not reveal a significant alteration of the G/F ratio with respect to control, as evaluated by western blot analysis (Fig. 3B, C). However, due to the poor subcellular resolution of the assay, we could not exclude a potential local defect at the synapse, where PRRT2 is mostly expressed. For this reason, we examined the intensity of 488-phalloidin as a readout of synaptic F-actin concentration. Hippocampal neurons were transduced at 7 DIV with mCherry-tagged Scramble, ShPRRT2 or Sh+rPRRT2 lentiviral vectors, fixed and processed for immunofluorescence at 14 DIV. Quantitative analysis revealed that the fluorescence distribution of phalloidin puncta colocalizing with the presynaptic marker Bassoon in PRRT2-silenced neurons was significantly shifted toward lower intensities as compared to the control, indicating a reduction in the synaptic F-actin content. The co-expression of rPRRT2 together with ShPRRT2 normalized the F-actin intensity to the control distribution (Fig. 3D, E).

### PRRT2 silencing in hippocampal neurons alters actin dynamics at synapses and affects spine density and morphology

**Fig. 4:**
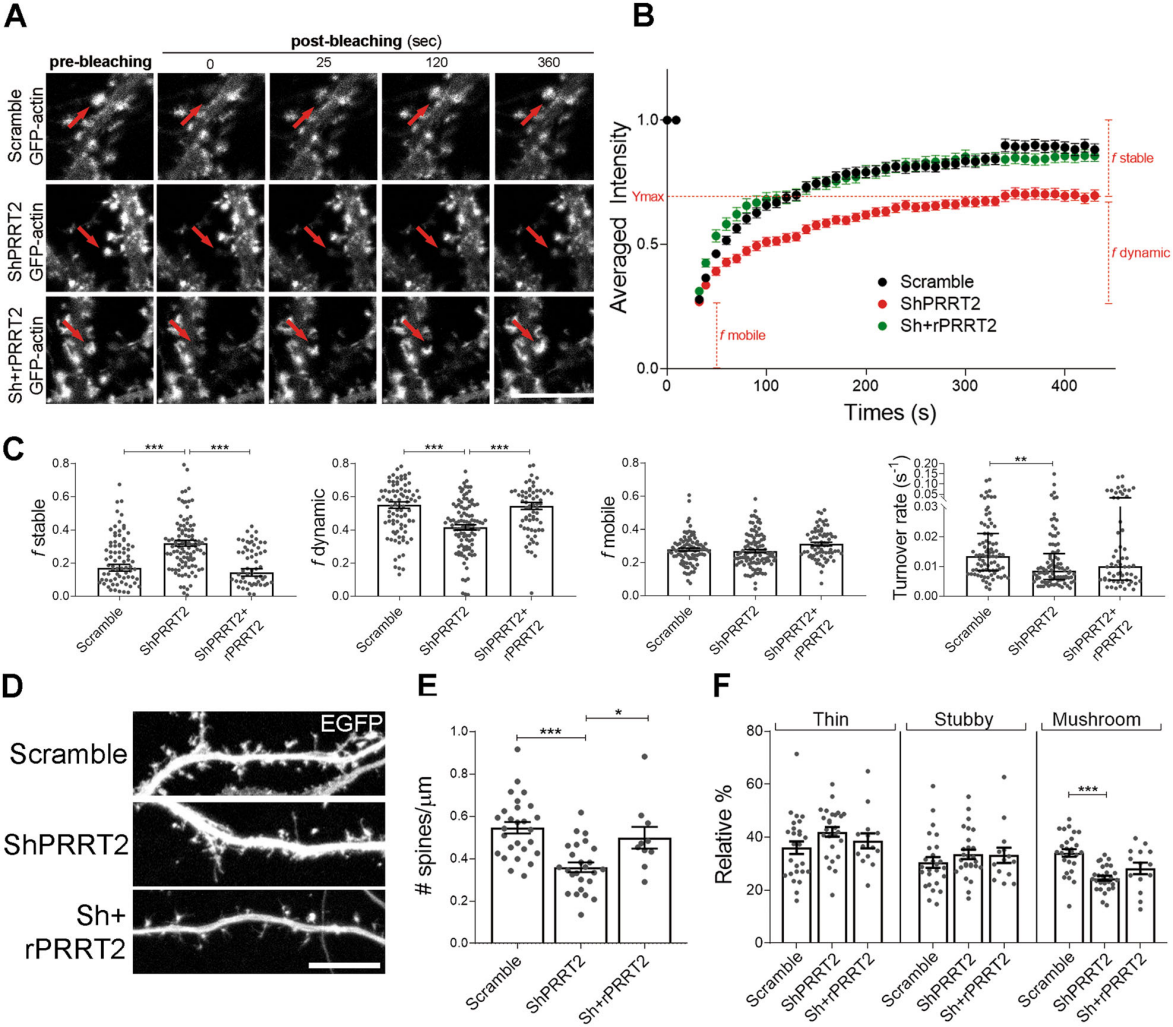
Knockdown of PRRT2 in hippocampal neurons disrupts F-actin polymerization and perturbs dendritic spine density and morphology. **A** Representative frames of prebleaching and postbleaching phases of EGFP-actin signals of live-imaged Scramble, ShPRRT2 or Sh+rPRRT2 transduced neurons. The regions of interest (ROI; diameter = 1.3 µm) are indicated by red arrows. Scale bar: 5 μm. **B** Quantitative analysis of the relative fluorescence intensity of EGFP-actin measured in the ROI over time, normalized to the mean signal measured in the prebleaching phase. Data are expressed as mean ± SEM for the fractions and median ± interquartile range for the turnover rate of n = number of spines from 4 independent experiments (Scramble = 92, ShPRRT2 = 103, Sh+rPRRT2 = 70). **C** Fraction of EGFP-actin in stable (*f* stable), dynamic (*f* dynamic) or mobile (*f* mobile) forms and the turnover rate of actin filaments in the absence or presence of PRRT2. In red are indicated the fractions referred to the ShPRRT2 curve. *f* stable: Scramble = 0.17 ± 0.02, ShPRRT2 = 0.32 ± 0.02, Sh+rPRRT2 = 0.14 ± 0.02; *f* dynamic: Scramble = 0.55 ± 0.02, ShPRRT2 = 0.42 ± 0.02, Sh+rPRRT2 = 0.54 ± 0.02; *f* mobile: Scramble = 0.28 ± 0.01, ShPRRT2 = 0.27 ± 0.01, Sh+rPRRT2 = 0.31 ± 0.01. Data are expressed as mean ± SEM of n = number of spines. One-way ANOVA/Bonferroni’s tests; **p < 0.01, ***p < 0.001. Median turnover rate (s^−1^): Scramble = 0.0135, ShPRRT2 = 0.0085, Sh+rPRRT2 = 0.0101; Kruskal–Wallis/Dunn’s tests: **p < 0.01. **D** Representative images of 14 DIV hippocampal neurons infected at 7 DIV with mCherry-tagged Scramble, ShPRRT2, or Sh + Sh-resistant PRRT2 (Sh+rPRRT2) lentiviruses. EGFP was transfected at 4 DIV to visualize cell profiles. Scale bar: 10 μm. **E** Quantitative analysis (means ± SEM) of the number of dendritic spines per μm of neurite (spine density) in neurons treated as in **D**, detected with the NeuronStudio software. Mean spine density ± SEM: Scramble = 0.55 ± 0.02, ShPRRT2 = 0.34 ± 0.03, Sh+rPRRT2 = 0.48 ± 0.05; *p* < 0.001 Scramble vs ShPRRT2, *p* < 0.05 ShPRRT2 vs Sh+rPRRT2. **F** Morphometric analysis of dendritic spines. The percent distribution of thin, stubby and mushroom spines is shown.: Scramble = 36 ± 2, ShPRRT2 = 42 ± 2, Sh+rPRRT2 = 38 ± 3; percentage of spines: Scramble = 30 ± 2, ShPRRT2 = 34 ± 2, Sh+rPRRT2 = 33 ± 3; percentage of spines: Scramble = 34 ± 1, ShPRRT2 = 24 ± 1, Sh+rPRRT2 = 28 ± 2; *p* < 0.001 Scramble vs ShPRRT2. Data in **E** and **F** are shown as means ± SEM of *n* = total number of neurons from 3 independent experiments (Scramble = 27, ShPRRT2 = 27, Sh+rPRRT2 = 14). One-way ANOVA/Bonferroni’s tests; *p < 0.05, ***p < 0.001.

In principle, the decreased synaptic F-actin content observed in PRRT2-silenced synapses could be ascribed to a local alteration of actin polymerization. To address this hypothesis, we performed fluorescence recovery after photobleaching (FRAP) on the synaptic EGFP-actin signal. Neurons were transfected with an EGFP-actin construct at 4 DIV and infected at 7 DIV with either mTurquoise-tagged Scramble or ShPRRT2 lentivirus or double infected with ShPRRT2-mTurquoise and rPRRT2-mCherry lentiviruses. Neurons were subjected to the FRAP protocol at 14–15 DIV. The average fluorescence recovery of EGFP-actin signal was followed over time after bleaching of a circular ROI positioned at a dendritic spine head (Fig. 4A). Interestingly, we found that PRRT2 silencing slowed down the EGFP-actin fluorescence recovery (Fig. 4B). The EGFP-actin fluorescence recovery curve is usually considered as being composed by three different fractions: *f* mobile, *f* dynamic and *f* stable^24,25,30^ and the turnover rate of actin filaments can be calculated as 1/τ where τ represent the timepoint at which the dynamic component reaches half the value of full recovery. By adopting this analysis, we determined that the general alteration in the recovery rate of actin signal in PRRT2-silenced neurons was due to a significant reduction of the dynamic fraction, which represents the polymerization of new actin filaments, and to a significant increase in the stable fraction, which represents actin structures with slower turnover that would require more time to recover. These alterations in the dynamic and stable fractions of EGFP-actin were completely rescued by re-expression of rPRRT2 (Fig. 4C). Together, these observations point to an impairment of actin turnover and stability in dendritic spines in the absence of PRRT2.

To verify whether the alteration of the actin polymerization could affect dendritic spine formation or maintenance, we analysed spine density and morphology. Primary hippocampal neurons were transfected with an EGFP plasmid at 4 DIV and infected at 7 DIV with mCherry-tagged Scramble or ShPRRT2 lentivirus or double infected with ShPRRT2 and rPRRT2-mCherry lentiviruses. Neurons were then analysed at 14 DIV, when synapses and dendritic spines have acquired full functional maturation. As compared to the Scramble control group, neurons lacking PRRT2 displayed a significant reduction in spine density that was completely rescued by co-expression of rPRRT2 in silenced neurons (Fig. 4D, E). In addition, we observed that downregulation of PRRT2 caused a selective decrease of mature mushroom-shaped spines (−10%), accompanied by a relative increase of immature (thin) ones (+6%) (Fig. 4F). The maturation state of the spines was partially normalized by co-expressing rPRRT2 in silenced neurons. To further challenge the ability of PRRT2 to influence spine development, we transduced wild-type neurons at 7 DIV with a lentiviral vector expressing either mCherry-tagged rPRRT2 or mCherry alone. While PRRT2 overexpression did not cause a significant alteration in spine density at 14 DIV (Supplementary Fig. 4A, B), the expression of PRRT2 reduced the relative abundance of mushroom spines (−7%), while increasing that of thin spines (+6%) (Supplementary Fig. 4C). The data confirm that loss of PRRT2 reduces the number of spines and support a direct participation of the protein in the control of their morphological maturation.

### The effects of PRRT2 silencing on the synaptic cytoskeleton and spine density are associated with an alteration of cofilin activity

**Fig. 5:**
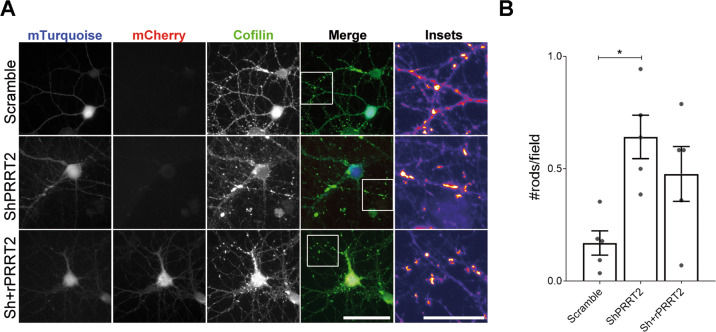
Knockdown of PRRT2 in hippocampal neurons induces the formation of cofilin-actin rods. **A** 7 DIV hippocampal neurons were infected with either mTurquoise-tagged Scramble or ShPRRT2 or co-infected with mTurquoise-ShPRRT2 and rPRRT2-mCherry lentiviral vectors. After 7 days (14 DIV), rods were stained using cofilin antibody and recognized as dense elongated bundles along neurites as shown in the insets. Scale bar: 20 μm (Insets: 25 μm). **B** Quantitative analysis of the rod density expressed as number of rods per field. Mean ± SEM n. rods/field: Scramble = 0.170 ± 0.05, ShPRRT2 = 0.641 ± 0.09, Sh+rPRRT2 = 0.477 ± 0.12. Data are expressed as mean ± SEM of the number of rods found in each experiment from five independent experiments. For each coverslip at least 20 random fields were acquired. One-way ANOVA/Bonferroni’s tests; *p < 0.05.

A fundamental regulator of actin dynamics within synapses is cofilin, an actin-binding protein that promotes actin remodelling^31,32^. To test whether PRRT2 silencing broadly impairs actin-related signalling pathways, we analysed the total level and phosphorylation state of cofilin and major upstream molecules involved in its activation by western blotting. However, we did not observe any overt alteration specific for PRRT2 silencing, and the decreases in Src phosphorylation and PAK expression were not rescued by PRRT2 overexpression (Supplementary Figs. 5 and 6). An imbalance between the actin concentration and cofilin activity is known to promote the accumulation of cofilin-actin aggregates along neurites, also defined “rods”^33^. Thus, we counted the number of cofilin rods in 14 DIV neurons infected at 7 DIV with mTurquoise-tagged Scramble or mTurquoise-tagged ShPRRT2 lentivirus, or double infected with ShPRRT2-mTurquoise and rPRRT2-mCherry lentiviruses. Hippocampal neurons silenced for PRRT2 accumulated a significantly higher number of cofilin rods as compared to the Scramble control group, while co-expression of rPRRT2 substantially rescued the phenotype (Fig. 5A, B).

### The synaptic morphological defects observed in PRRT2-depleted neurons are attributable to a hyperactivation of cofilin

**Fig. 6:**
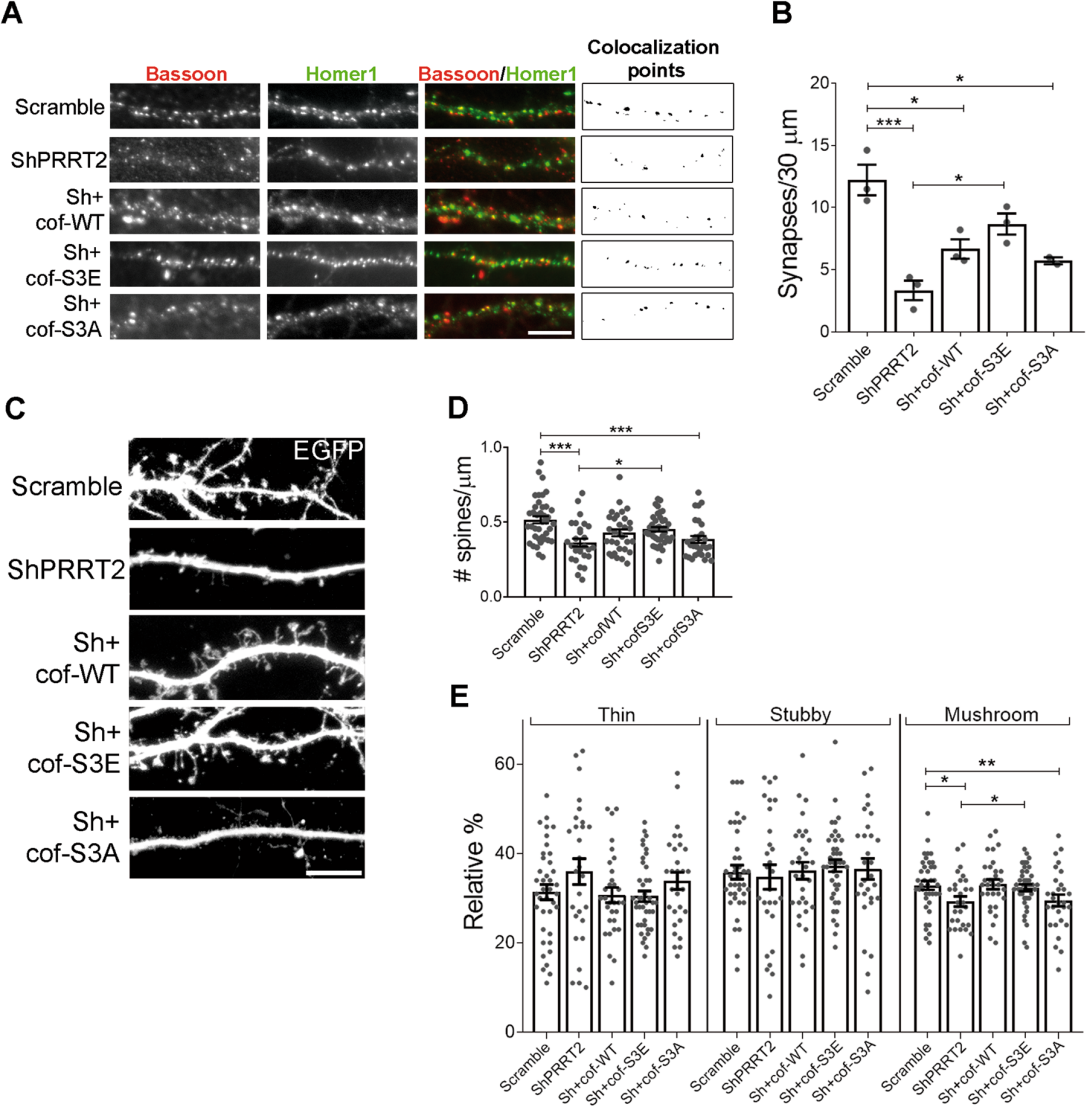
Expression of a constitutively inactive form of cofilin in hippocampal neurons rescues the phenotype induced by PRRT2 knockdown. **A** Representative dendrites of hippocampal neurons infected at 7 DIV with mCherry-tagged Scramble or ShPRRT2 in the presence or absence of wild-type (cof-WT), dephospho-mimetic (S3A) or phospho-mimetic (S3E) cofilin and analysed at 14 DIV. Synaptic boutons were identified by double immunostaining for Bassoon (red) and Homer1 (green). The colocalization panel highlights 0.1–1 µm^2^ double-positive puncta (black) corresponding to synapses. Scale bar: 10 µm. **B** Quantitative analysis of synaptic puncta counted on 30-µm dendrite segments starting from the cell body in neurons treated as in **A**. Mean synapses/30 μm ± SEM: Scramble = 12.22 ± 1.22, ShPRRT2 = 3.34 ± 0.78, Sh+cof-WT = 6.67 ± 0.77, Sh+cof-S3E = 8.68 ± 0.85, Sh+cof-S3A = 5.72 ± 0.28; *p* < 0.001 Scramble vs ShPRRT2, *p* < 0.05 Scramble vs Sh+cof-WT, *p* < 0.05 Scramble vs Sh+cof-S3A, *p* < 0.05 ShPRRT2 vs Sh+cof-S3E. Data are expressed as means ± SEM from *n* = 3 independent experiments. One-way ANOVA/Bonferroni’s tests; *p < 0.05, ***p < 0.001. **C** 14 DIV dendrites of hippocampal neurons transfected with EGFP at DIV 4 to visualize dendritic spines. At 7 DIV neurons were infected as reported in **A**. Scale bar: 10 μm. **D** Quantitative analysis of spine density and **E** morphometric analysis of dendritic spines. Mean spine density ± SEM: Scramble = 0.51 ± 0.02, ShPRRT2 = 0.36 ± 0.03, Sh+cof-WT = 0.42 ± 0.02, Sh+cof-S3E = 0.45 ± 0.01, Sh+cof-S3A = 0.38 ± 0.02; *p* < 0.001 Scramble vs ShPRRT2, *p* < 0.001 Scramble vs Sh+cof-S3A, *p* < 0.05 ShPRRT2 vs Sh+cof-S3E. Relative % thin spines: Scramble = 31 ± 2, ShPRRT2 = 36 ± 3, Sh+cof-WT = 31 ± 2, Sh+cof-S3E = 31 ± 1, Sh+cof-S3A = 34 ± 2; relative % stubby spines: Scramble = 36 ± 2, ShPRRT2 = 35 ± 3, Sh+cof-WT = 36 ± 2, Sh+cof-S3E = 37 ± 1, Sh+cof-S3A = 37 ± 2; relative % mushroom spines: Scramble = 33 ± 1, ShPRRT2 = 29 ± 1, Sh+cof-WT = 33 ± 1, Sh+cof-S3E = 32 ± 1, Sh+cof-S3A = 30 ± 2; *p* < 0.05 Scramble vs ShPRRT2, *p* < 0.01 Scramble vs Sh+cof-S3A, *p* < 0.05 ShPRRT2 vs Sh+cof-S3E. Data in **D** and **E** are shown as means ± SEM of *n* = total number of neurons from 5 independent experiments (total number of neurons: Scramble = 39, ShPRRT2 = 28, Sh+cof-WT = 31, Sh+cof-S3E = 41, Sh+cof-S3A = 29). One-way ANOVA/Bonferroni’s tests; *p < 0.05, **p < 0.01, ***p < 0.001.

To verify whether the unbalanced cofilin activity was responsible for the reduction in synaptic density observed in PRRT2-silenced neurons, we tested whether the overexpression of constitutively active or inactive cofilin was able to rescue the phenotype. Cofilin activity is negatively regulated by phosphorylation on serine 3, thus we exploited a cofilin phospho-mimetic mutant (cof-S3E, constitutively inactive) and a cofilin dephospho-mimetic mutant (cof-S3A, constitutively active). We double-infected hippocampal neurons with a lentivirus expressing mCherry-tagged ShPRRT2 together with a lentivirus expressing either WT, S3E or S3A human cofilin. Synaptic density was analysed by immunofluorescence with pre and postsynaptic markers to unambiguously identify synaptic contacts. The analysis revealed that the density of excitatory synapses was decreased by about 50% in PRRT2 KD neurons, in-line with published evidence^4^. Importantly, only neurons double infected with Sh and cof-S3E showed a complete normalization of the number of synapses, while only a partial rescue was observed with cof-WT and S3A overexpression (Fig. 6A, B).

To assess whether a perturbation of cofilin activity in PRRT2-silenced neurons was also critical for the defects observed in dendritic spines, we measured the density and morphology of the spines in double-infected neurons. Co-infection with cof-S3E, but not with cof-WT or cof-S3A, was able to significantly rescue the ShPRRT2 phenotype (Fig. 6C, D). Consistently, the reduction in the frequency of mushroom-shaped spines induced by the loss of PRRT2 was statistically normalized only by co-expression of cof-S3E (Fig. 6E). These results suggest that the synaptic morphological defects observed in neurons lacking PRRT2 are attributable to a hyperactivation of cofilin that can be counteracted by expression of the constitutively inactive form cofilin-S3E.

### The morphological recovery induced by cofilin-S3E does not rescue the impaired synaptic transmission of PRRT2-depleted neurons

**Fig. 7:**
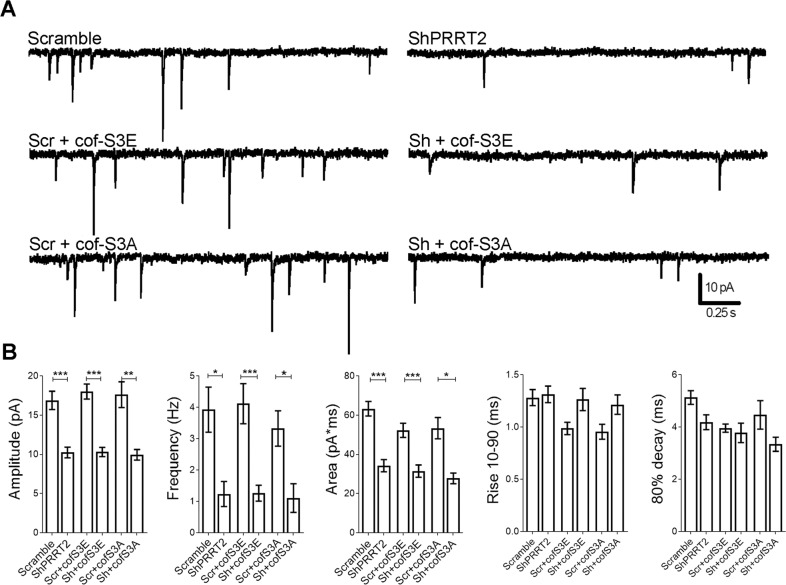
The expression of cofilin phosphomutants in PRRT2-silenced hippocampal neurons does not modify spontaneous synaptic transmission. Low-density hippocampal neurons were infected at 7 DIV with mCherry-tagged Scramble or ShPRRT2 lentivirus in combination with cof-S3E-mCherry or cof-S3A-mCherry. At 14 DIV, mEPSCs were recorded in whole-cell patch-clamp with holding potential of −70 mV. **A** Representative traces of mEPSCs from all phenotypes tested. **B** Analysis of mEPSCs. Amplitude, frequency, area, rise 10–90% and 80% decay of mEPSCs are expressed as mean ± SEM of *n* = number of cells (Scramble = 11, ShPRRT2 = 14, Scr+cof-S3E = 21, Sh+cofS3E = 20, Scr+cof-S3A = 8, Sh+cofS3A = 7 from 3 independent experiments). Amplitude (pA) mean ± SEM: Scramble = 16.90 ± 1.17, ShPRRT2 = 10.24 ± 0.69, Scr+cof-S3E = 18.02 ± 0.96, Sh+cofS3E = 10.30 ± 0.59, Scr+cof-S3A = 17.62 ± 1.65, Sh+cofS3A = 9.94 ± 0.68, *p* < 0.001 Scramble vs ShPRRT2, *p* < 0.001 Scr+cofS3E vs Sh+cof-S3E, *p* < 0.01 Scr+cofS3A vs Sh+cof-S3A; frequency (Hz): Scramble = 3.93 ± 0.72, ShPRRT2 = 1.24 ± 0.40, Scr+cof-S3E = 4.12 ± 0.64, Sh+cofS3E = 1.26 ± 0.25, Scr+cof-S3A = 3.36 ± 0.56, Sh+cofS3A = 1.11 ± 0.46, *p* < 0.05 Scramble vs ShPRRT2, *p* < 0.001 Scr+cofS3E vs Sh+cof-S3E, *p* < 0.05 Scr+cofS3A vs Sh+cof-S3A; area (pA*ms): Scramble = 63.28 ± 3.72, ShPRRT2 = 34.24 ± 3.13, Scr+cof-S3E = 52.27 ± 3.65, Sh+cofS3E = 31.44 ± 3.14, Scr+cof-S3A = 53.37 ± 5.44, Sh+cofS3A = 27.75 ± 2.75; *p* < 0.001 Scramble vs ShPRRT2, *p* < 0.001 Scr+cofS3E vs Sh+cof-S3E, *p* < 0.05 Scr+cofS3A vs Sh+cof-S3A; rise 10–90 (ms): Scramble = 1.28 ± 0.08, ShPRRT2 = 1.31 ± 0.08, Scr+cof-S3E = 0.94 ± 0.05, Sh+cofS3E = 1.26 ± 0.11, Scr+cof-S3A = 0.96 ± 0.07, Sh+cofS3A = 1.22 ± 0.09; 80% decay (ms): Scramble = 5.13 ± 0.26, ShPRRT2 = 4.18 ± 0.28, Scr+cof-S3E = 3.96 ± 0.16, Sh+cofS3E = 3.78 ± 0.37, Scr+cof-S3A = 4.47 ± 0.55, Sh+cofS3A = 3.34 ± 0.27. Statistical analysis was conducted with Student’s *t*-test (Scramble vs ShPRRT2 couples); *p < 0.05, **p < 0.01, ***p < 0.001.

Finally, we evaluated whether the expression of cof-S3E was able to rescue synaptic function that is strongly impaired in PRRT2-silenced neurons^4^. Hippocampal neurons were infected at 7 DIV with mCherry-tagged either Scramble or ShPRRT2 lentiviruses in combination with cof-S3E or cof-S3A. Cells were then analyzed at 14 DIV using whole-cell patch-clamp to record miniature excitatory postsynaptic currents (mEPSCs). In agreement with previous data, silencing of PRRT2 significantly reduced the amplitude, frequency and charge of mEPSCs as compared to Scramble-transduced neurons. However, co-expression of either cof-S3A or S3E failed to normalize any of these parameters (Fig. 7A, B), indicating that the morphological recovery induced by cof-S3E is not paralleled by a functional rescue.

## Discussion

In the present study, we employed both non-neuronal cells and hippocampal neurons in vitro to reveal a novel functional interaction between PRRT2 and the actin-remodelling machinery.

Our data indicate that the ectopic expression of PRRT2 reduces cell motility and intercellular space of migrating cells in a wound-healing assay, and promotes cell clustering in an aggregation assay, thus indicating that PRRT2 promotes cell–cell adhesion. PRRT2 ectopic expression also reduces the fraction of pTyr^397^FAK-positive focal adhesions, without altering their total number. Since FAK phosphorylation is required to recruit the endocytic adaptor protein dynamin^34^, it is possible that PRRT2 specifically influences focal adhesion disassembly and thus cell-ECM adhesive properties, ultimately altering the migration capability. Cell-ECM and cell–cell adhesion complexes are functionally linked and clearly involved in the modulation of the actin cytoskeleton^35^. In-line with this, we additionally show that the ectopic expression of PRRT2 induces the formation of actin-rich filopodia in several cell lines and modifies cell shape, supporting an unexpected action of PRRT2 on actin regulation. Interestingly, the N-terminal intracellular portion of the protein seems to be necessary to elicit these effects, possibly by being engaged in specific interactions with the adhesive machinery.

In neurons, the actin cytoskeleton plays a crucial role during synaptogenesis: the first event that will eventually lead to the formation of a synaptic contact is indeed the protrusion of a dendritic actin-rich filopodium which, upon contact with a presynaptic counterpart, will give rise to a postsynaptic dendritic spine^36^. Adhesive molecules are actively involved in the recognition and stabilization of pre/postsynaptic contacts, particularly by instructing the actin cytoskeleton to drive the morphological modelling of the synapse^37,38^. The actin cytoskeleton is fundamental in mature synapses as well, where it sustains the function of both the pre and postsynaptic compartments. We show that PRRT2 deletion in hippocampal neurons in culture leads to a reduction of synaptic F-actin content and to a local dysregulation of actin polymerization dynamics at synapses, as assessed by the analysis of phalloidin fluorescence intensity at synaptic puncta and by FRAP experiments at single spines. This is paralleled by an impairment of spine extension and maturation. By combining the information gathered in cells lines and in hippocampal neurons, we thus hypothesize that, during early synaptogenic events, PRRT2 participates in conveying adhesive messages from adhesion molecules contacting the ECM and nearby cells to the actin cytoskeleton, in order to guide proper synaptic maturation.

A high number of actin-binding proteins operate to tightly regulate every aspect of actin dynamics. Among them, cofilin has been extensively characterized because of its key role at the synaptic level, particularly in the morphogenesis of dendritic spines^39,40^. The silencing of cofilin in neurons induces spine loss and shrinkage^32,41^, reminiscent of the phenotype observed in PRRT2-silenced neurons. A local perturbation of cofilin activity induced by its hyperactivation and/or by an unbalanced stoichiometry between cofilin and actin is known to lead to the formation of cofilin-actin rods. These structures sequester and redistribute actin and cofilin in bundles along neurites rather than at synapses, thus disrupting normal actin dynamics and causing synaptic dysfunction^33,42^. In PRRT2-silenced neurons, the reduction of the synaptic concentration of actin filaments probably leads to an imbalance of the local cofilin:actin ratio. Consistently, we observed an increase in cofilin-actin rods in the absence of PRRT2. Although we did not detect any overt alteration in the phosphorylation of cofilin, a local hyperactivation of cofilin selectively at synapses is plausible. Accordingly, the expression of a constitutively inactive phospho-mimetic mutant of cofilin was able to counteract virtually all the structural synaptic defects induced by PRRT2 depletion, in terms of synapse number, spine density and morphology. In contrast, the overexpression of inactive cofilin did not normalize the defective spontaneous transmission of PRRT2-silenced neurons. This result is consistent with the idea that the impairment of neurosecretion observed in the absence of PRRT2 depends on disruption of the Ca^2+^-sensing machinery and disturbance in cellular excitability, which involve molecular interactors distinct from those involved in actin dynamics^4,5^.

Our results indicate that the structural synaptic defects observed in PRRT2-silenced neurons are not a secondary event caused by defective neurotransmitter release, and that PRRT2 exerts a role in synapse formation and maintenance involving the regulation of actin dynamics that is functionally separated from its role in neurotransmitter release. This interpretation is in agreement with the previously reported finding that synaptogenesis and spine morphogenesis are preserved even in the complete absence of neurotransmitter release, as shown in neurons depleted for Munc-13 and/or 18^43,44^. Along this line, SNAP-25-silenced neurons, in addition to obvious abnormalities in neurotransmitter release caused by defective SNARE-complex assembly, display defects in dendritic spine morphogenesis that are specifically due to a mislocalization of p140Cap, an interactor of SNAP-25 whose function converges on actin cytoskeleton remodeling^45^. This evidence further testifies that these two aspects—synaptic structure and neurotransmitter release—rely at least in part on independent mechanisms.

In conclusion, we demonstrated a novel role of PRRT2 in modulating synapse morphogenesis through the regulation of the cofilin-actin axis. Thanks to its dual role, PRRT2 may thus act at the interface between the modulation of synaptic activity and the regulation of synapse morphology, which impinge on distinct signaling pathways and have to be strictly coordinated during synaptic formation and for the proper scaling of synaptic function in plasticity phenomena^12^. For these reasons, we propose that the loss of both these independent functions of PRRT2 contributes to the phenotypes of PRRT2-mutated patients, and that would probably be necessary to simultaneously restore both in order to obtain a full synaptic recovery.

## Supplementary information

Supplementary Figure legends

Supplementary procedures

Supplementary Figure 1

Supplementary Figure 2

Supplementary Figure 3

Supplementary Figure 4

Supplementary Figure 5

Supplementary Figure 6
