# Supplementary material for: Proline-rich transmembrane protein 2 (PRRT2) regulates the actin cytoskeleton during synaptogenesis
Source: Cell Death Dis. 2020 Oct 14;11(10):856. doi: 10.1038/s41419-020-03073-w (PMC7560900; doi:10.1038/s41419-020-03073-w)
Supplement: Supplementary file 1 — Supplementary Figure legends [file 41419_2020_3073_MOESM1_ESM.docx]

**Supplementary Figure Legends**

**Supplementary Figure 1.** **PRRT2 ectopic expression induces the formation of filopodia in NIH 3T3 and HEK293T cells.** Representative confocal images of NIH 3T3 (A) or HEK293T (B) cells transfected with either EGFP (control) or PRRT2-EGFP (green). Cells were fixed and incubated with 568-phalloidin to stain F-actin (red) and with Hoechst 33342 solution (blue) to stain nuclei. The high magnification merge images on the right (insets) highlight cell protrusive elements. Scale bar: 40 μm (insets = 10 μm) for (A) and 20 μm (insets = 10 μm) for (B).

**Supplementary Figure 2. The overexpression of PRRT2-EGFP does not affect the general activation of Cdc42 and Rac1 GTPases in HeLa cells.** (A) To validate the protocol, protein lysate from non transfected cells was incubated with GDP (negative control) or GTPγS (positive control) prior to the incubation with PAK-DBT beads. As shown by the corresponding lane, GTPγS irreversibly bound most of the Cdc42 and Rac1 molecules present in the sample, which was thus precipitated with the beads. In contrast, the saturation with GDP replaced most of the GTP bound to the Cdc42 and Rac1, which were weakly able to bind the beads. (B) As a control to check the responsiveness of the Rac1 signalling pathway a group of cells were serum starved for 24 h to reduce the amount of active Rac1. After this, some cells were treated with 10 ng/ml of EGF, which is a potent activator of Rac1, for 2 min prior to the protocol. (C) PRRT2-EGFP, Cdc42 and Rac1 protein levels of HeLa cells transfected with either EGFP or PRRT2-EGFP. For each sample the input, the pulldown fraction (PD) and the unbound fraction (UNB) were loaded. (D) Quantitative densitometric analysis of protein levels. Total protein levels, corrected by the GAPDH level of each sample, and the ratios between the pulldown fraction (active, GTP-bound) and the input protein levels are normalized to the EGFP sample, which is set to 1. Cdc42/GAPDH: EGFP = 1, PRRT2-EGFP = 1.14 ± 0.12; PD/Input Cdc42: EGFP = 1, PRRT2-EGFP = 1.44 ± 0.25; Rac1/GAPDH: EGFP = 1, PRRT2-EGFP = 1.33 ± 0.21; PD/Input Rac1: EGFP = 1, PRRT2-EGFP = 1.08 ± 0.18. Data are means ± SEM of n = 6 independent experiments.Student’s t-test.

**Supplementary Figure 3.** Silencing and rescue of endogenous PRRT2 in primary neurons at different MOI of lentivirus infection. Representative immunoblot of PRRT2, mCherry and Caspase-3 in 14 DIV hippocampal neurons infected at 7 DIV with increasing MOI of mCherry-tagged Scramble, ShPRRT2 or Sh+rPRRT2 lentiviruses. The asterisk (*) indicates PRRT2-mCherry fusion protein. GAPDH was used as loading control to normalize band intensities for each protein of interest.

**Supplementary Figure 4.** **PRRT2 overexpression does not alter synapse density.** (A) Representative images of dendrites of 14 DIV neurons infected at 7 DIV with either mCherry or PRRT2-mCherry lentivirus. EGFP was transfected at 4 DIV to decorate cell profiles. Scale bar: 10 μm. (B) Quantitative analysis of the number of dendritic spines per μm of neurite (spine density) in neurons treated as in (A) using the NeuronStudio software. Mean spine density ± SEM: mCherry = 0.43 ± 0.06 vs PRRT2 = 0.55 ± 0.09. (C) Morphometric analysis of the relative abundance of thin, stubby and mushroom dendritic spines. Relative % thin spines: mCherry = 34, PRRT2-mCherry = 40; relative % stubby spines: mCherry = 28, PRRT2-mCherry = 30; relative % mushroom spines: mCherry = 37, PRRT2-mCherry = 30. Data are means ± SEM of n = 11 and 13 mCherry and PRRT2-mCherry neurons respectively, from 2 independent experiments (= 13). Student’s *t*-test; *p<0.05.

**Supplementary Figure 5. PRRT2 downregulation in neurons does not grossly perturb the cofilin activation signalling pathway.** (A) Scheme of the cofilin-activation signalling pathway. SRC and FAK are phosphorylated upon signalling deriving from integrin and/or cadherin receptors at the plasma membrane. Once the SRC-FAK complex is recruited, it activates several pathways including Rac1 and Cdc42 GTPases. GTP-bound Rac1 and Cdc42 activate the p21-activated kinase (PAK) that, through LIM kinase (LIMK), induces phosphorylation of cofilin and its inhibition. (B) Representative immunoblot of PRRT2, pSer^3^Cofilin, Cofilin, pTyr^416^SRC, SRC, pThr^423^PAK, PAK, pTyr^397^FAK, FAK protein levels in 14 DIV hippocampal neurons infected at 7 DIV with mCherry-tagged Scramble, ShPRRT2, or Sh+rPRRT2. The asterisk (*) indicates PRRT2-mCherry fusion protein. GAPDH was used as loading control to normalize band intensities for each protein of interest. (C) Densitometric analysis of protein levels. Data were normalized on the mean of non-infected (NI) samples. The changes in Src phosphorylation or PAK expression following PRRT2 silencing are not rescued by PRRT2 overexpression. pSRC/SRC: Scramble = 1.20 ± 0.07, ShPRRT2 = 0.59 ± 0.04, Sh+rPRRT2 = 0.73 ± 0.03, p<0.001 Scramble vs ShPRRT2 and vs Sh+rPRRT2; SRC/GAPDH: Scramble = 0.80 ± 0.05, ShPRRT2 = 0.82 ± 0.11, Sh+rPRRT2 = 0.82 ± 0.10; pFAK/FAK: Scramble = 1.07 ± 0.30, ShPRRT2 = 1.42 ± 0.27, Sh+rPRRT2 = 1.19 ± 0.10; FAK/GAPDH: Scramble = 0.59 ± 0.10, ShPRRT2 = 0.40 ± 0.02, Sh+rPRRT2 = 0.42 ± 0.01; pPAK/PAK: Scramble = 0.64 ± 0.06, ShPRRT2 = 0.69 ± 0.18, Sh+rPRRT2 = 0.88 ± 0.27; PAK/GAPDH: Scramble = 1.25 ± 0.14, ShPRRT2 = 0.84 ± 0.07, Sh+rPRRT2 = 0.94 ± 0.08, p<0.05 Scramble vs ShPRRT2; pCOF/COF: Scramble = 0.72 ± 0.13, ShPRRT2 = 0.77 ± 0.11, Sh+rPRRT2 = 0.74 ± 0.09; COF/GAPDH: Scramble = 1.09 ± 0.14, ShPRRT2 = 0.84 ± 0.16, Sh+rPRRT2 = 0.83 ± 0.13. Data are expressed as mean ± SEM of n>3 independent experiments. One-way ANOVA/Bonferroni’s tests; *p<0.05, ***p<0.001.

**Supplementary Figure 6. The activation of Cdc42 and Rac1 GTPases is not affected by PRRT2 knock-down in hippocampal neurons.** (A) To validate the protocol, protein lysate from untreated hippocampal neurons was incubated with GDP (negative control) or GTPγS (positive control) prior to the incubation with PAK-DBT beads. As shown by the corresponding lane, GTPγS irreversibly bound most of the Cdc42 and Rac1 molecules present in the sample, which was thus precipitated with the beads. In contrast, the saturation with GDP replaced all the GTP bound to the Cdc42 and Rac1, which were not able to bind the beads. (B) PRRT2, Cdc42 and Rac1 protein levels of 11 DIV hippocampal neurons infected at 7 DIV with mCherry-tagged Scramble, ShPRRT2 or Sh+rPRRT2. For each sample the input, the pulldown fraction (PD) and the unbound fraction (UNB) were loaded. (C) Quantitative densitometric analysis of protein levels. Total protein levels, corrected by the GAPDH level of each sample, and the ratios between the pulldown fraction (active, GTP-bound) and the total protein levels of the input are normalized to the Scramble sample set to 1. Cdc42/GAPDH: Scramble = 1, ShPRRT2 = 1.14 ± 0.15, Sh+rPRRT2 = 1.11 ± 0.09; PD/Input Cdc42: Scramble = 1, ShPRRT2 = 1.47 ± 0.33, Sh+rPRRT2 = 1.01 ± 0.18; Rac1/GAPDH: Scramble = 1, ShPRRT2 = 1.19 ± 0.20, Sh+rPRRT2 = 0.94 ± 0.07; PD/Input Rac1: Scramble = 1, ShPRRT2 = 1.18 ± 0.09, Sh+rPRRT2 = 0.99 ± 0.09. Data are means ± SEM of n = 4 independent experiments for Cdc42, and n = 3 for Rac1. One-way ANOVA/Bonferroni’s tests.
