# Supplementary material for: Proline-rich transmembrane protein 2 (PRRT2) regulates the actin cytoskeleton during synaptogenesis
Source: Cell Death Dis. 2020 Oct 14;11(10):856. doi: 10.1038/s41419-020-03073-w (PMC7560900; doi:10.1038/s41419-020-03073-w)
Supplement: Supplementary file 2 — Supplementary procedures [file 41419_2020_3073_MOESM2_ESM.docx]

**Supplementary Procedures**

**Cdc42/Rac1 activation assay**

Cdc42/Rac1 activation assay was performed using the “Rac1 pull-down activation assay biochem” kit (Cytoskeleton #BK035) according to manufacturer’s instructions. Briefly, cells were washed once with ice cold PBS and lysed in lysis buffer containing 50 mM Tris pH 7.5, 10 mM MgCl_2_, 0.5 mM NaCl, 2% Igepal) supplemented with 1:100 protease inhibitors. Cells were scraped and collected. An aliquot of lysate was conserved for protein content quantification. A second aliquot was saved as “input” fraction for western blot quantification of total Rac1 and Cdc42. The remaining of samples was quickly frozen in liquid nitrogen and then stored at -80 °C for at least one day. Protein content was quantified with the bicinchoninic acid assay (BCA; Thermo Scientific Waltham, Massachusetts, USA #23228) according to manufacturer’s instructions. Frozen lysates were thawed in a 25 °C water bath. Positive and negative controls were prepared from non transfected cells or non-infected hippocampal lysates, supplemented with 150 mM EDTA and incubated with GTPγS (final concentration: 200 μM) for the positive control or with GDP (final concentration: 1 mM) for the negative control at RT under rotation for 15 min. The reaction was stopped by transferring samples on ice and adding 1:100 stop buffer provided in the kit. An equal volume of each sample was incubated at RT for 1 h with agarose beads conjugated with PAK-PBD domain, which are able to bind only GTP-bound Cdc42 and Rac1. Beads were isolated by centrifugation at 3 000 x g at 4 °C for 1 min and then the supernatant (containing the GDP-bound inactive form of the proteins) was removed and stored as the “unbound fraction”. Beads were resuspended in wash buffer (25 mM Tris pH 7.5, 30 mM MgCl_2_, 40 mM NaCl) followed by a 3 min centrifuge at 3 000 x g at 4 °C. After supernatant removal, pelleted beads were resuspended in Laemmli buffer 1X, heated for 2 min at 90 °C in order to elute the sample and then stored at -80 °C until use. Western blotting was performed as described below, by loading equal volumes of input, pulled-down and unbound fractions.

**Cell lysis, SDS-PAGE and Western blot analysis**

Neurons were quickly lysed with 1X Laemmli buffer (LB; 20 mM Tris-HCl pH 6.8, 2 mM EDTA, 2% SDS, 10% glycerol, 2% β-mercaptoethanol and 0.01% bromophenol blue), by adding the proper volume directly on the cells. When possible, samples were readily loaded on SDS-PAGE, otherwise they were stored at -80 °C until use.

Equal volumes of protein samples were subjected to SDS-PAGE (polyacrylamide gel electrophoresis) using the Standard Vertical Gel Electrophoresis unit (Hoefer, San Francisco, CA, USA) or precast NuPAGE 4-12% bis-tris gels (Invitrogen #NP0321). Samples were loaded after heating at 90 °C for 5 min. Markers were used as standards to extrapolate the molecular weight of the analysed protein samples (Biorad SDS-page standard high range #1610303, SDS-page standard low range #1610304, precision plus protein kaleidoscope #1610375 Hercules, CA, USA). Electrophoretic separation was performed in running buffer 1X (50 mM Tris-HCl, 384 mM gycine, 0.1% SDS) at a voltage of 80 V in the stacking gel and 150 V in the running gel. NuPAGE gels separation was performed in MOPS (Life Technologies, Monza, Italy #NP0001) at a voltage of 80 V in the stacking gel and 110 V in the running gel. The separated proteins were transferred to a nitrocellulose membrane (0.45 μm; GE Healthcare, Buckinghamshire, UK #10600002). Transfer was performed overnight in transfer buffer (25 mM Tris-HCl, 192 mM glycine, 20% methanol) at 250 mA at 4°C. The nitrocellulose membrane was stained with Ponceau Red (Sigma #P7170) to assess proper transfer. The membrane was cut horizontally in correspondence of the appropriate molecular weights and used for western blotting.

Blocking of the membranes was performed in 5% milk in TBST (Tris-Base saline-Tween: 150 mM NaCl, 20 mM Tris-HCl, pH 7.4, 0.05% Tween 20) for 1 h at RT. Primary antibodies were appropriately diluted in 5% milk in TBST and incubated for either 2 h at RT or overnight at 4 °C. Membranes were washed three times in TBST to eliminate primary antibody in excess. Secondary horseradish peroxidase (HRP) conjugated antibodies were diluted 1:10 000 in 5% milk in TBST and incubated for 1 h at RT. Membranes were washed three times in TBST to remove secondary antibodies in excess. Detection was performed with the enhanced chemiluminescence reaction (ECL Prime; GE Healtcare #16929851). Signals were detected with Chemidoc (Biorad Chemidoc MP Imaging system).

Densitometric quantification was performed with ImageLab software and data analysis was performed with Excel (Microsoft, Redmond, WA, USA). The amount of phosphorylation of a protein of interest was analysed loading the same sample on two separate gels in parallel and considering the ratio between the phosphorylated and total forms normalized on their own GAPDH.

List of antibodies used for western blot protocol:

- Actin, Sigma #A5441 Mouse; Monoclonal, 1:1 000

- PRRT2, Sigma #HPA014447 Rabbit; Polyclonal, 1:1 000

- Rac1, BD Biosciences #610650 Mouse; Monoclonal, 1:1 000

- Cdc42, Cell Signaling #2462 Rabbit; Polyclonal, 1:500

- GAPDH, Cell Signaling #2118 Rabbit; Polyclonal, 1:10 000

- dsRED, Clonotech #632496 Rabbit; Polyclonal, 1:1 000

- Caspase-3, Cell signalling #D3R6Y Rabbit; Polyclonal, 1:1 000

- pTyr^416^SRC, Cell signalling #2101 Rabbit; Polyclonal, 1:1 000

- SRC, Cell signaling #2108 Rabbit; Polyclonal, 1:1 000

- pThr^423^PAK, Cell Signaling #2601 Rabbit; Polyclonal, 1:500

- PAK, Santa Cruz #8801 Rabbit; Polyclonal, 1:1 000

- pTyr^397^FAK, ThermoFisher #44-6246 Rabbit; Polyclonal, 1:1 000

- FAK, Millipore #05-537 Mouse; Monoclonal, 1:1 000

- pSer^3^Cofilin, Cell Signaling #3313 Rabbit; Polyclonal, 1:1 000

- Cofilin, Abcam #54532 Mouse; Monoclonal, 1:500
