# Supplementary figures and images for: Proline-rich transmembrane protein 2 (PRRT2) regulates the actin cytoskeleton during synaptogenesis
Source: Cell Death Dis. 2020 Oct 14;11(10):856. doi: 10.1038/s41419-020-03073-w (PMC7560900; doi:10.1038/s41419-020-03073-w)

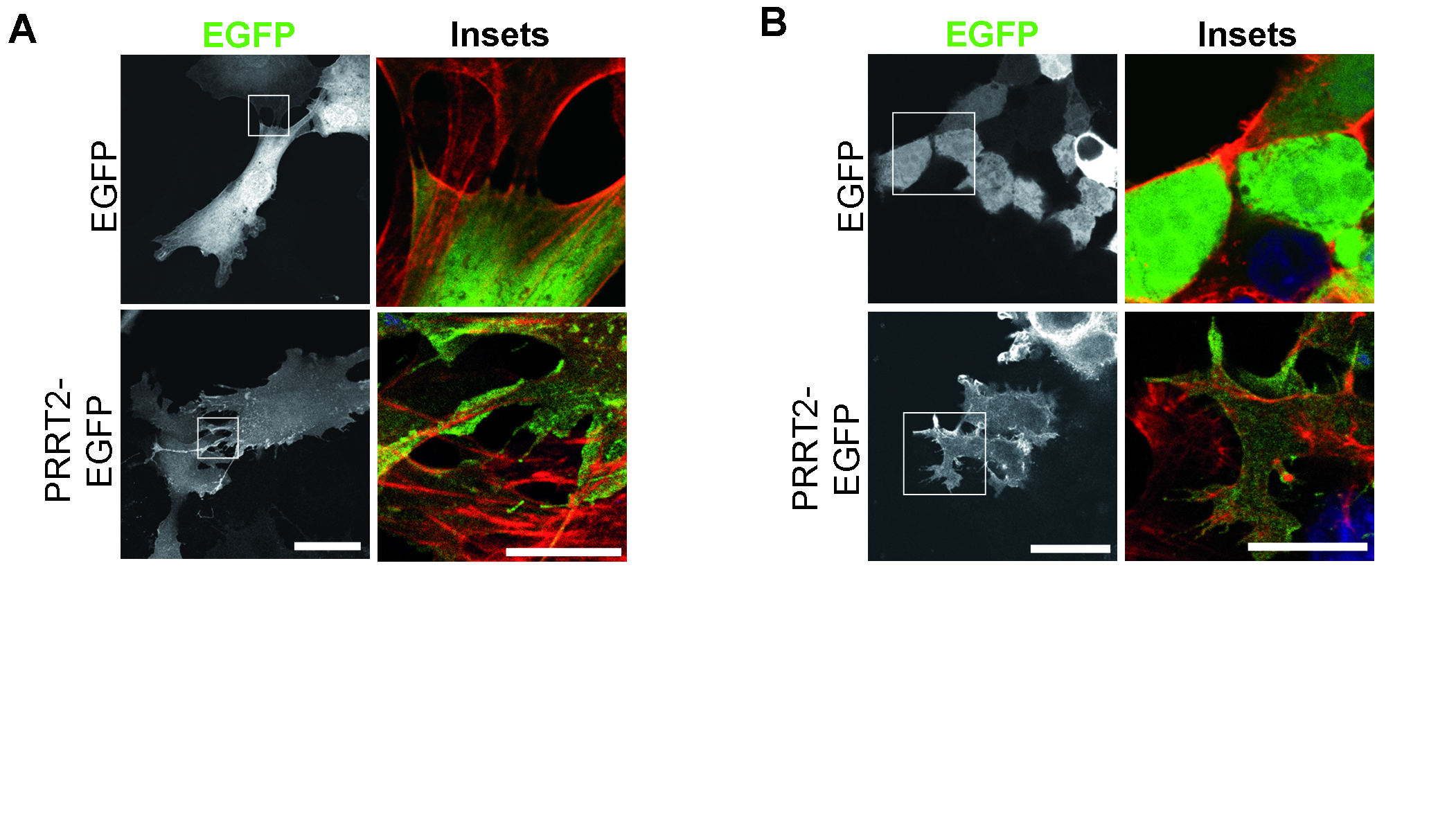

Supplement: Supplementary file 3 — Supplementary Figure 1 [file 41419_2020_3073_MOESM3_ESM.tif]

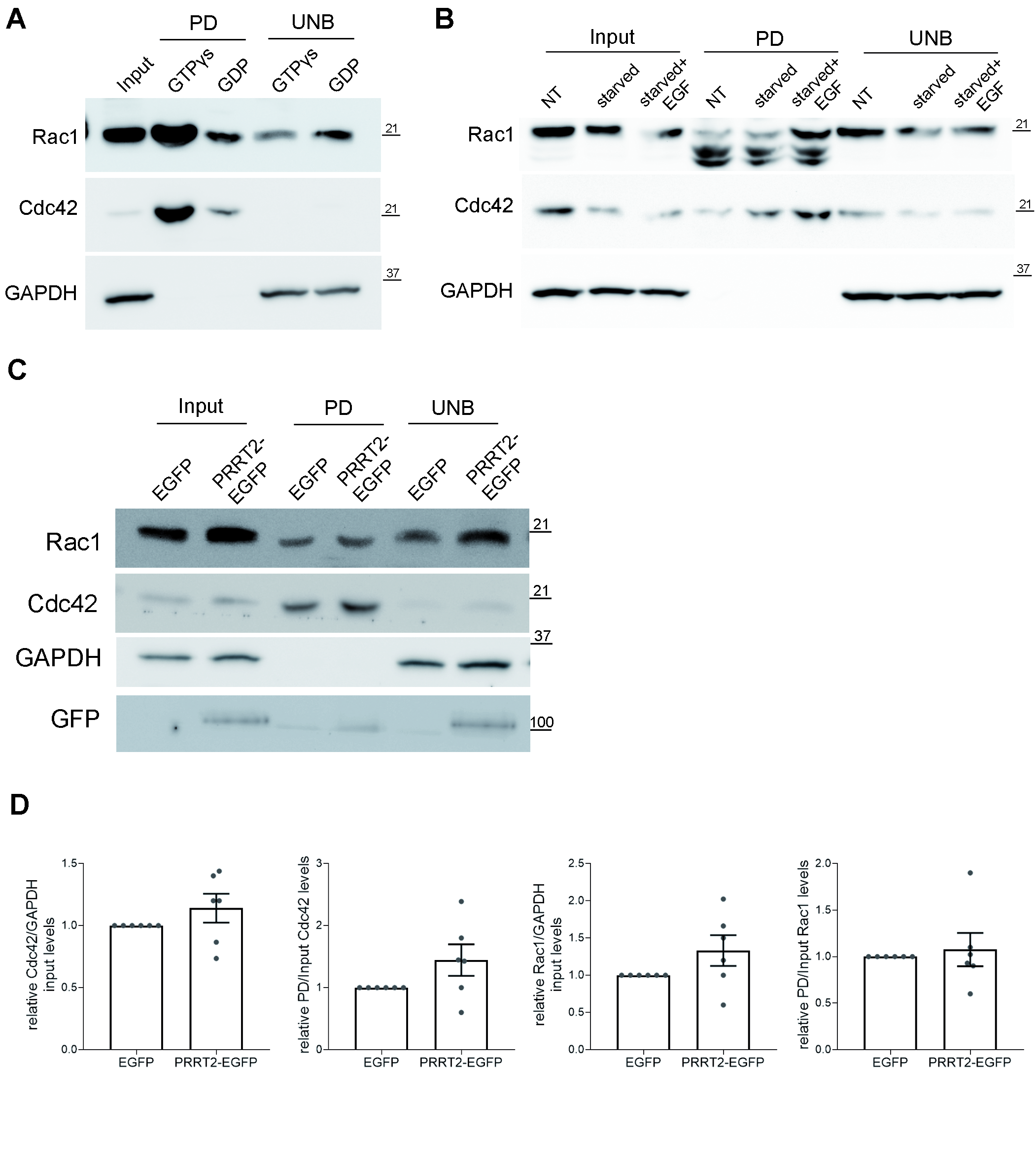

Supplement: Supplementary file 4 — Supplementary Figure 2 [file 41419_2020_3073_MOESM4_ESM.tif]

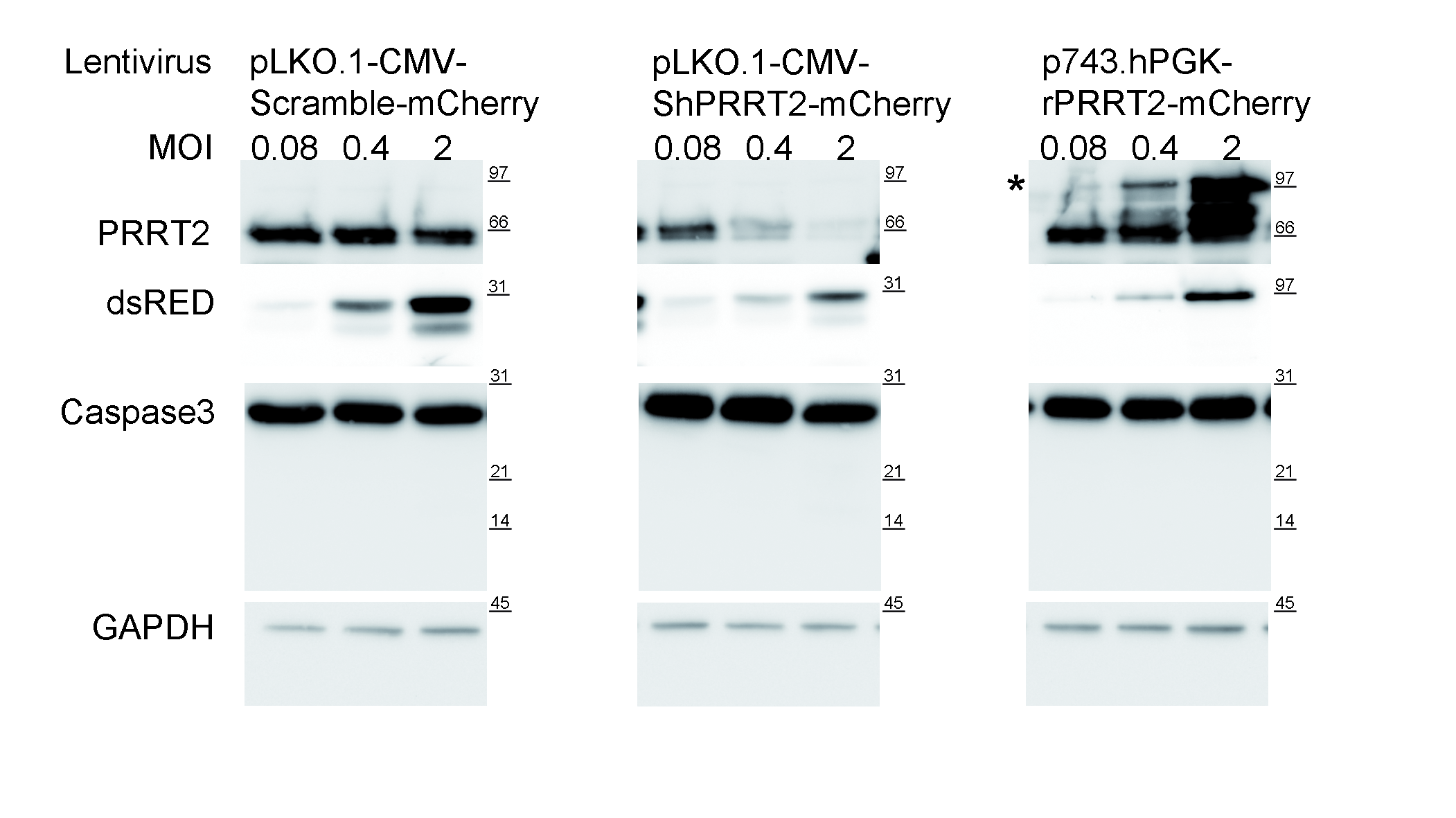

Supplement: Supplementary file 5 — Supplementary Figure 3 [file 41419_2020_3073_MOESM5_ESM.tif]

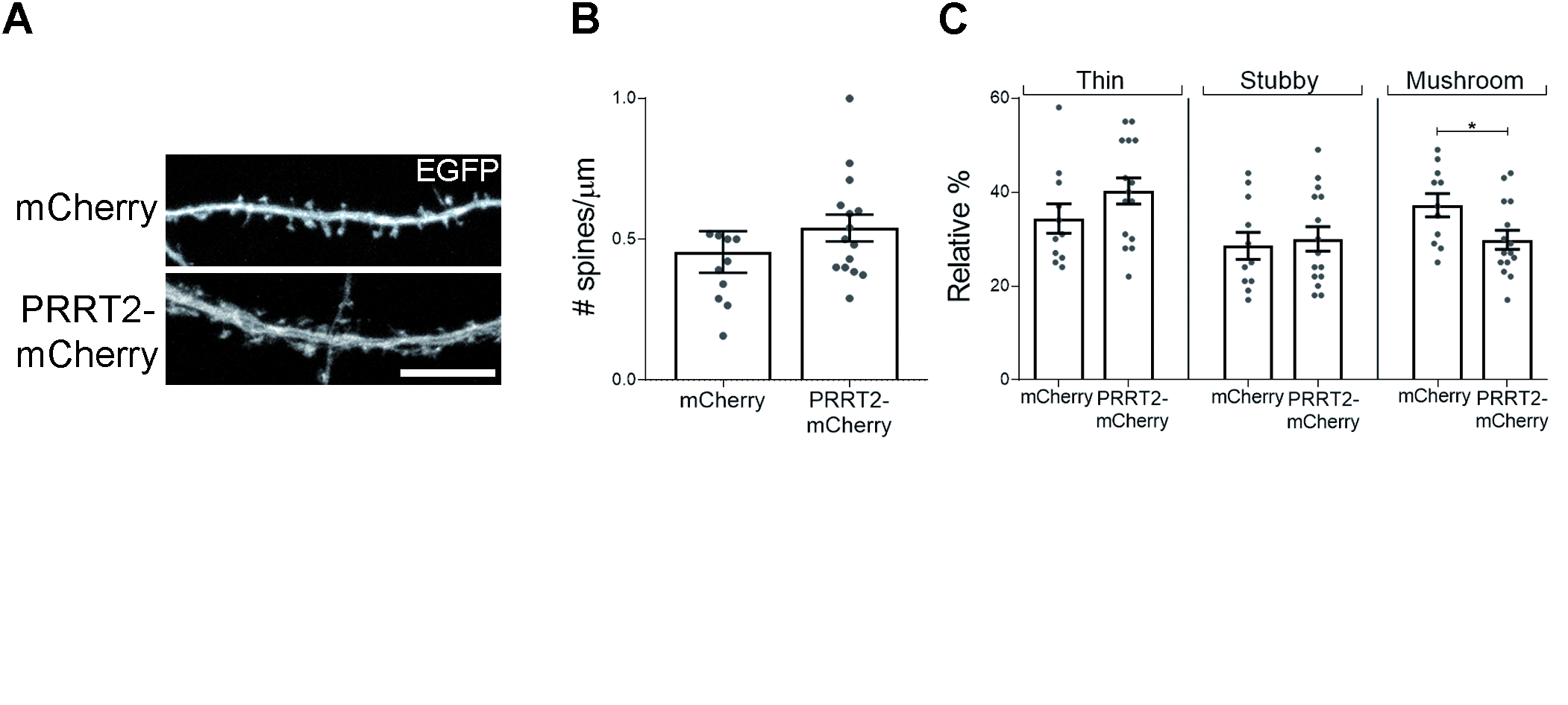

Supplement: Supplementary file 6 — Supplementary Figure 4 [file 41419_2020_3073_MOESM6_ESM.tif]

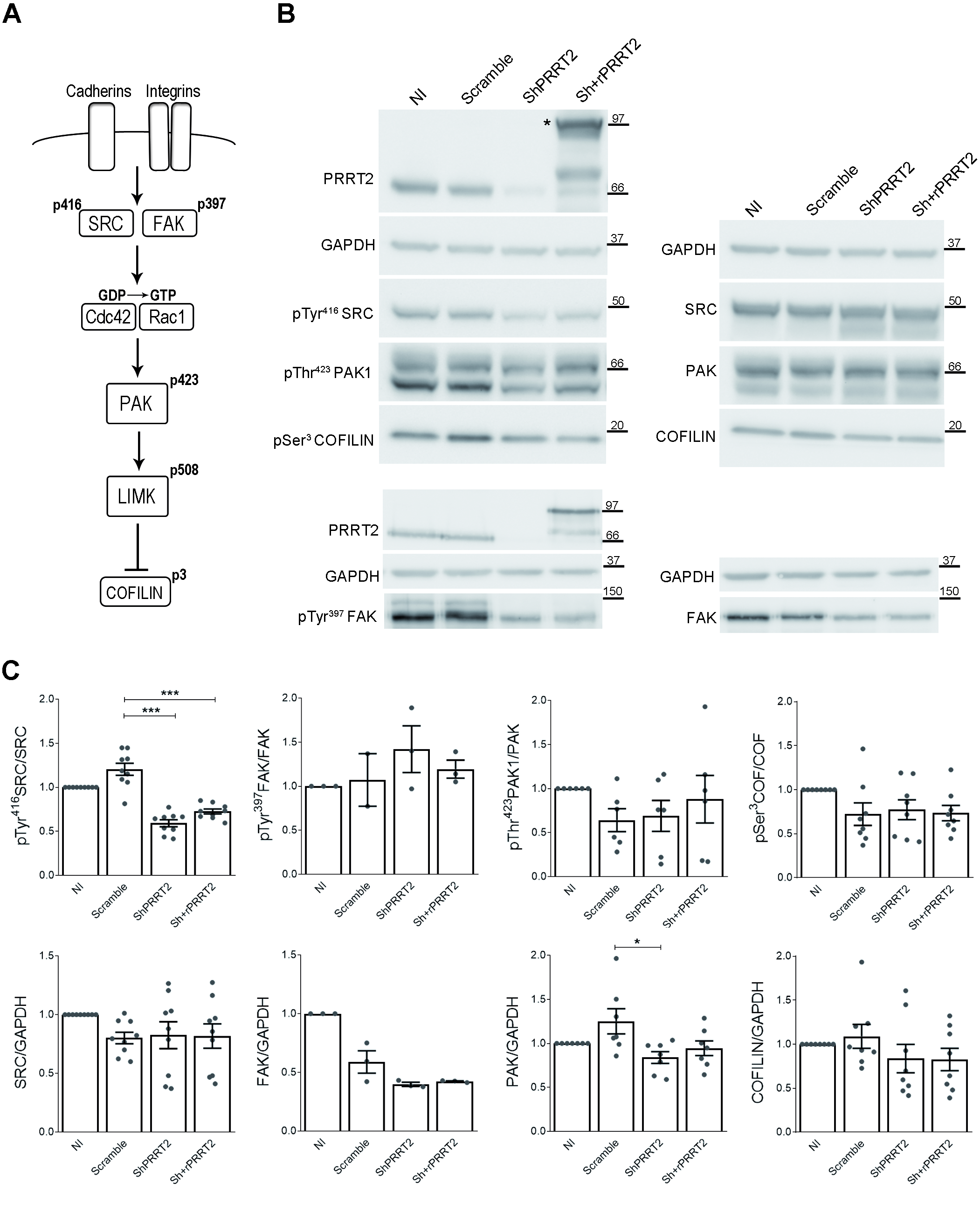

Supplement: Supplementary file 7 — Supplementary Figure 5 [file 41419_2020_3073_MOESM7_ESM.tif]

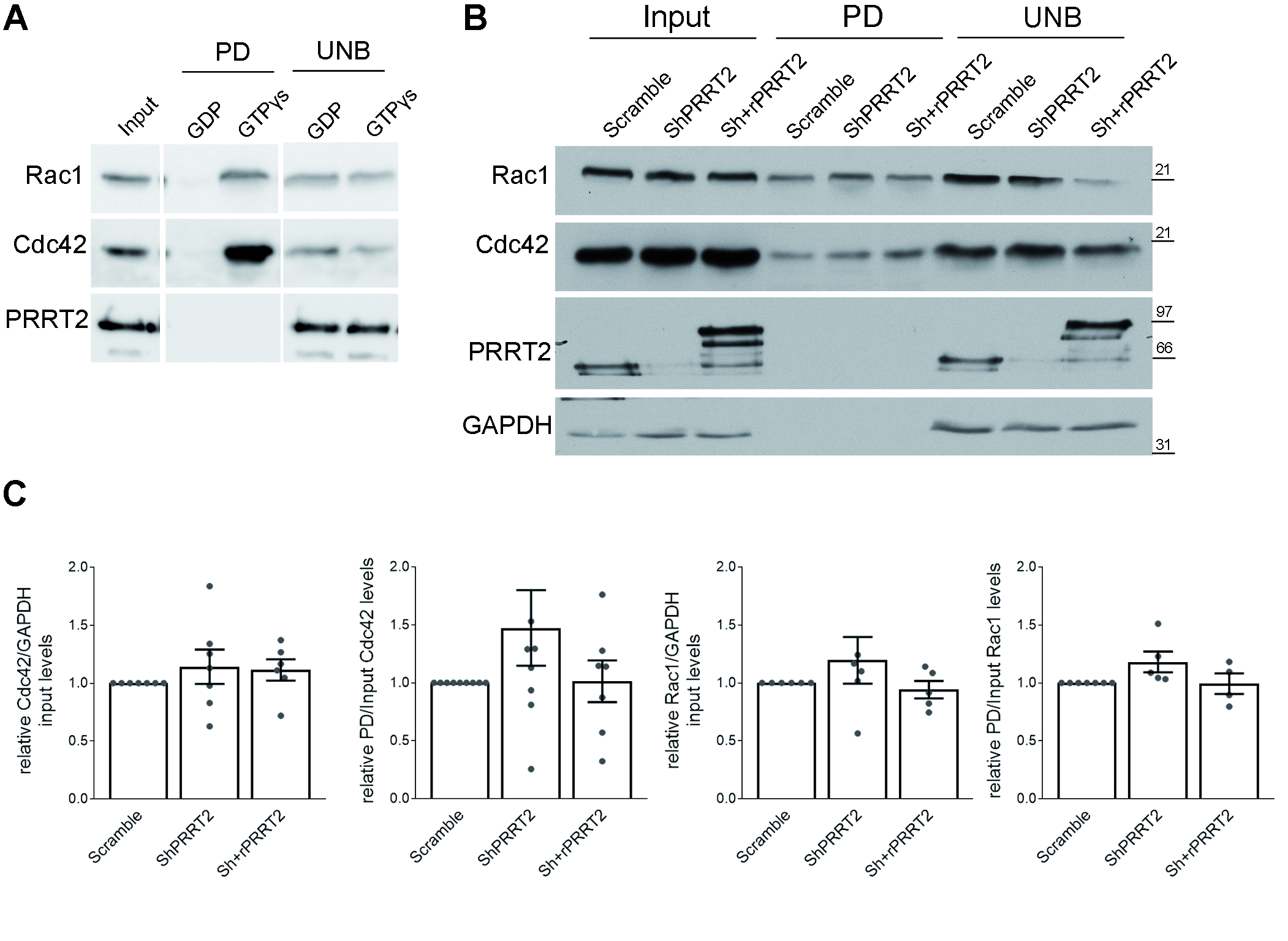

Supplement: Supplementary file 8 — Supplementary Figure 6 [file 41419_2020_3073_MOESM8_ESM.tif]
